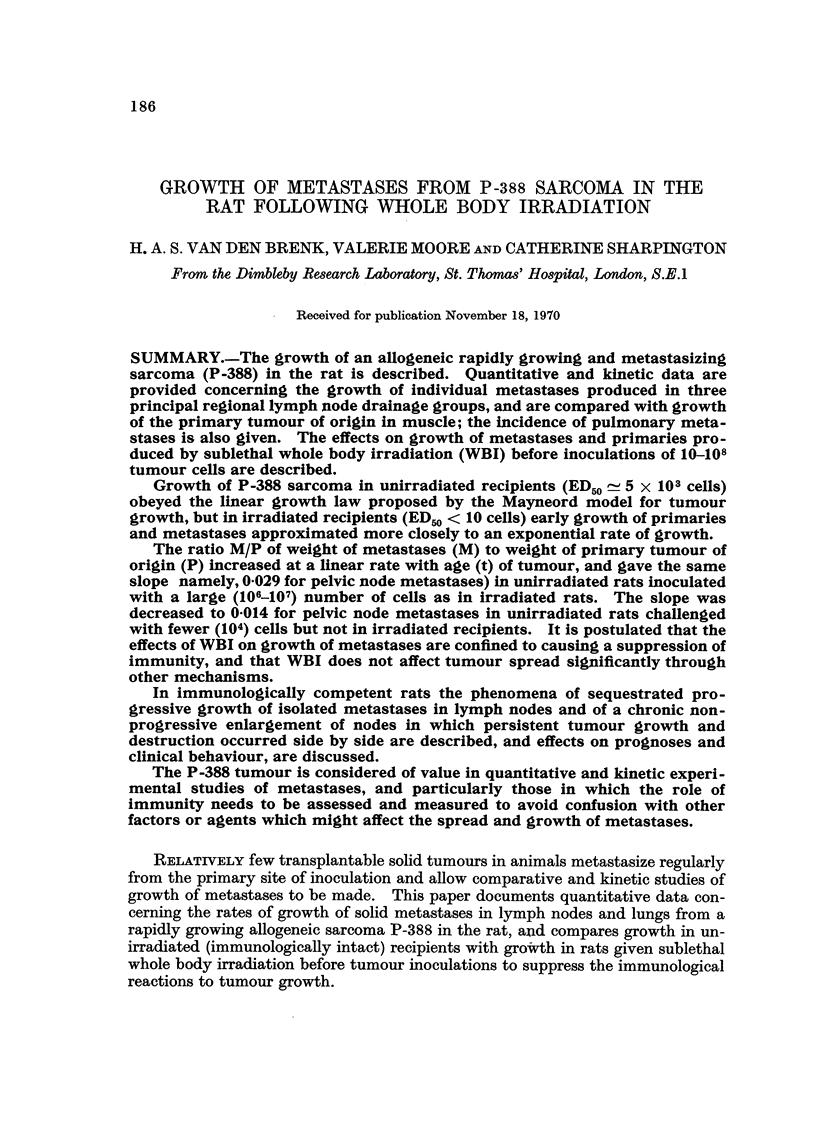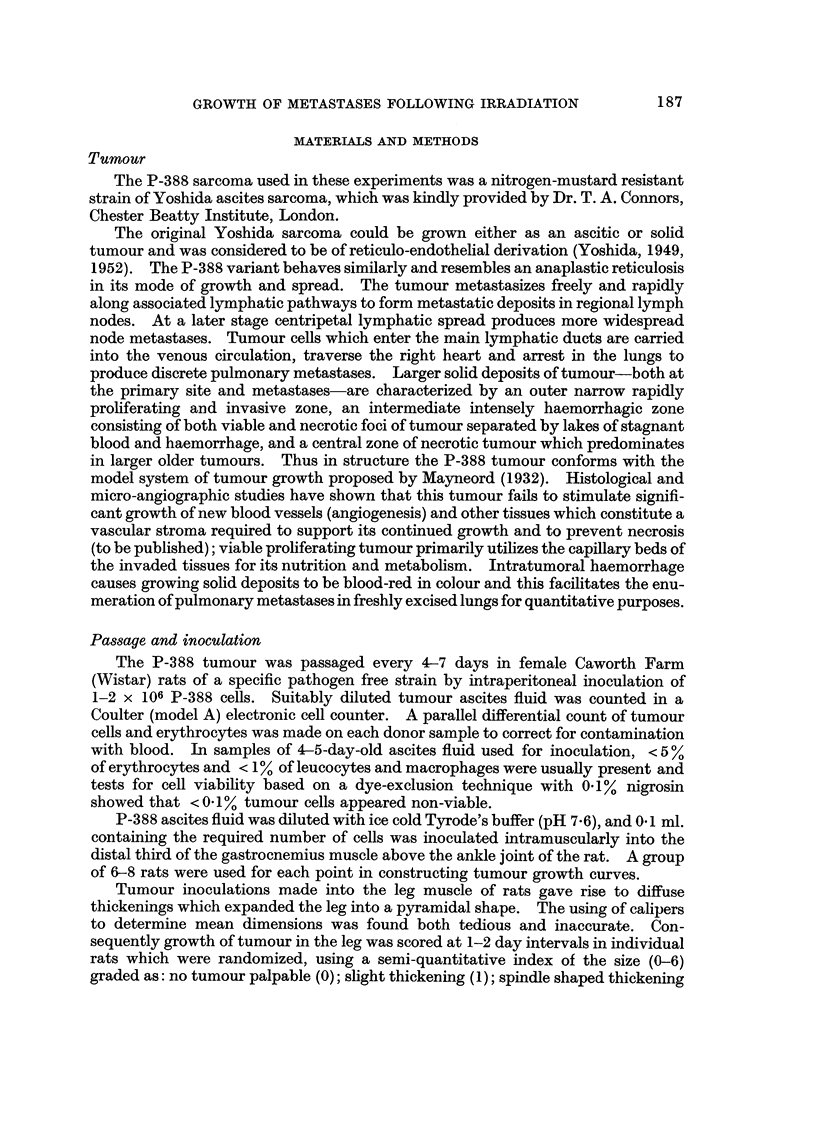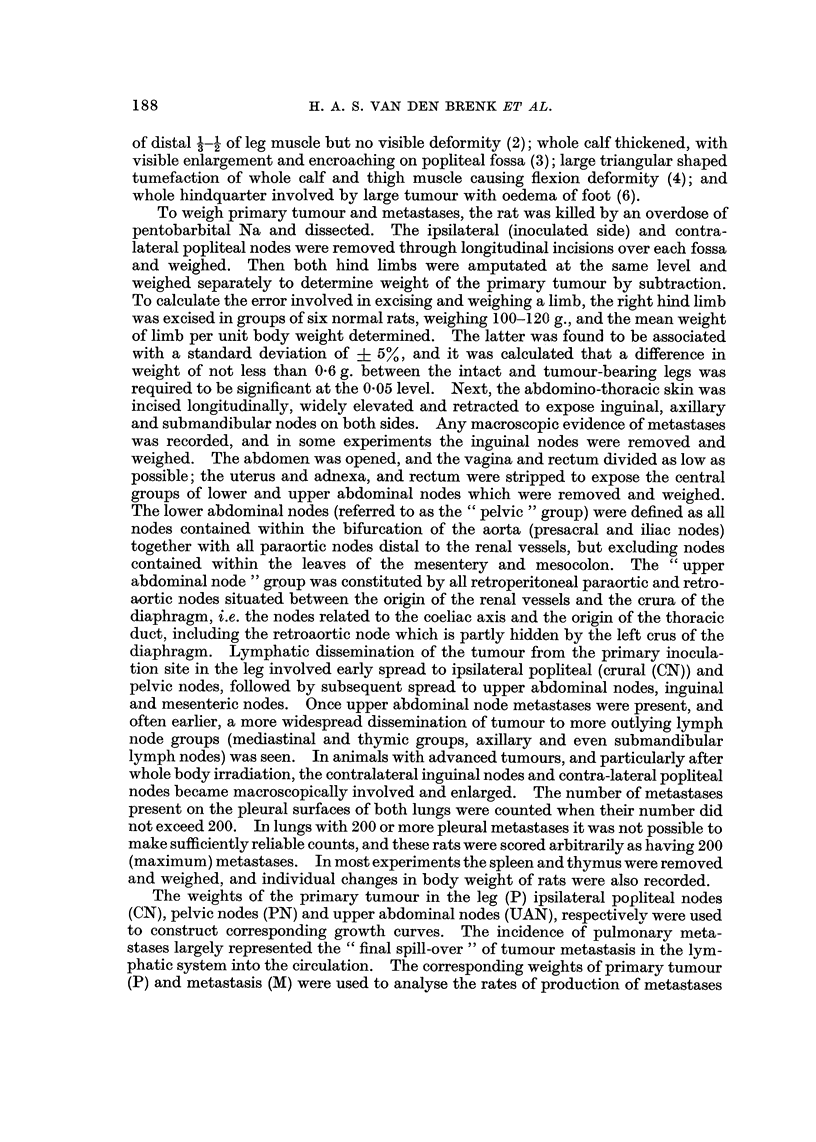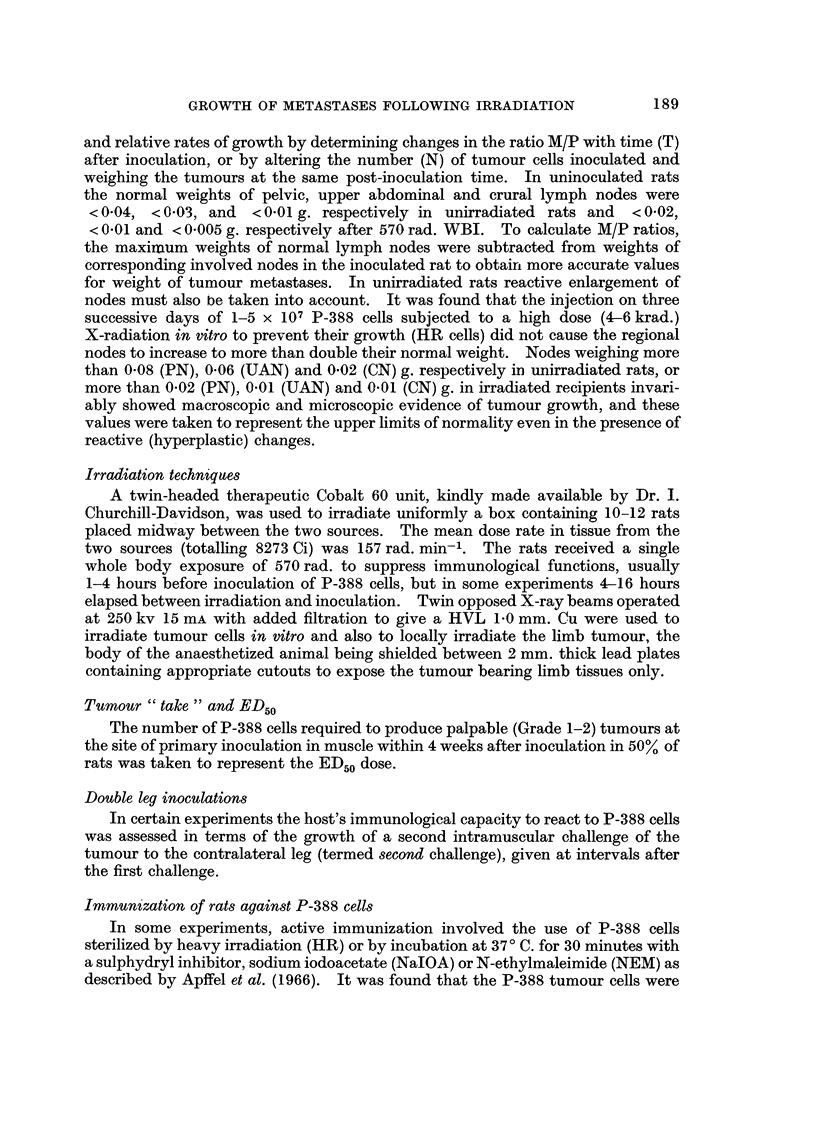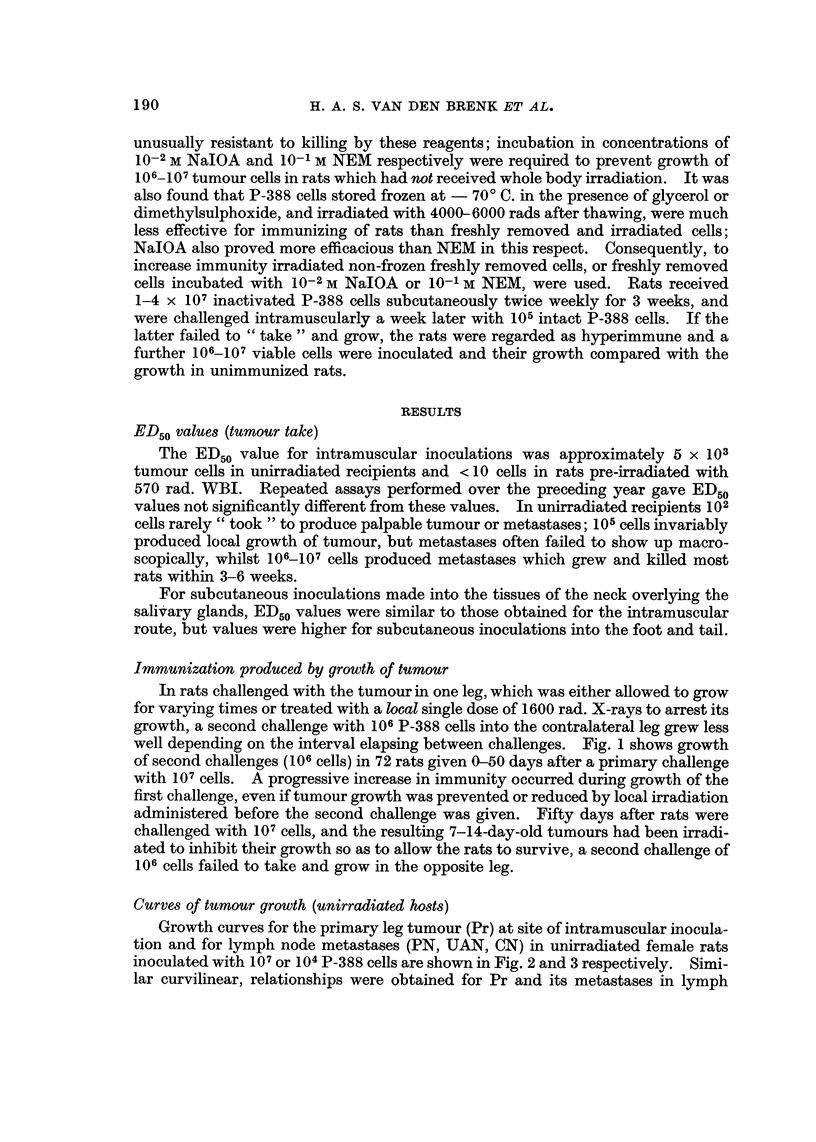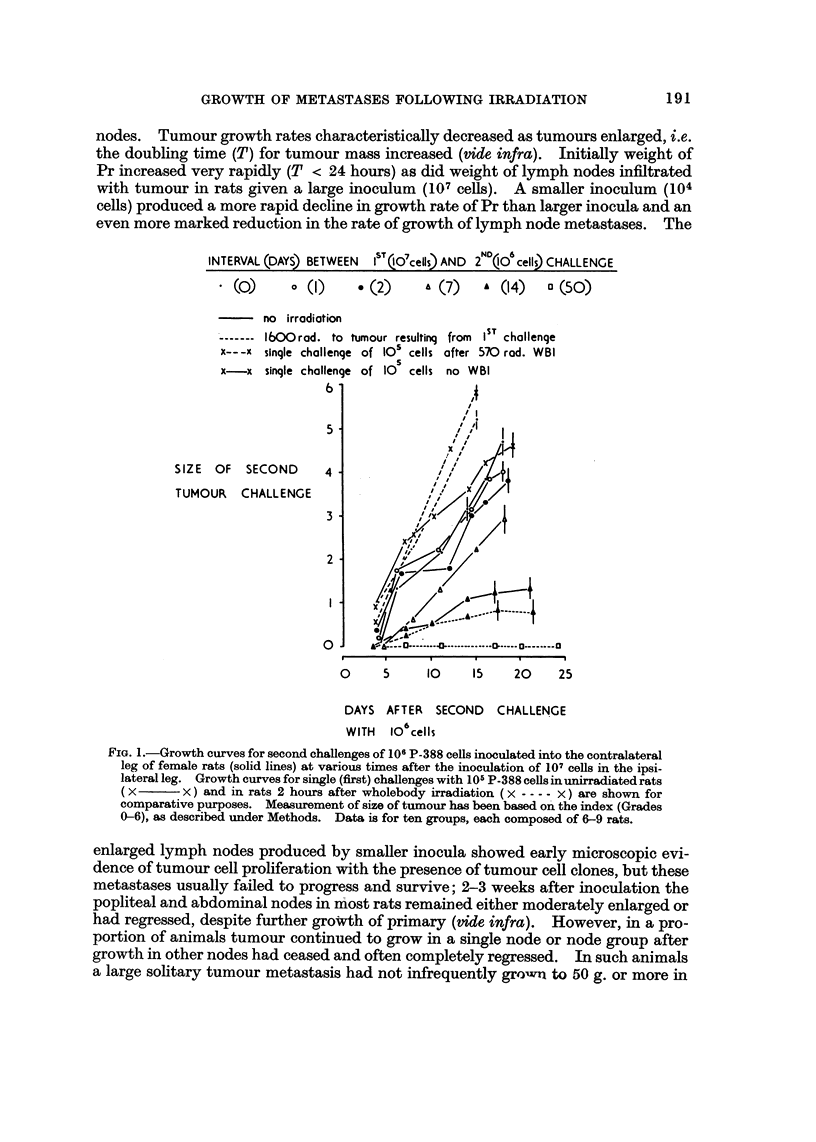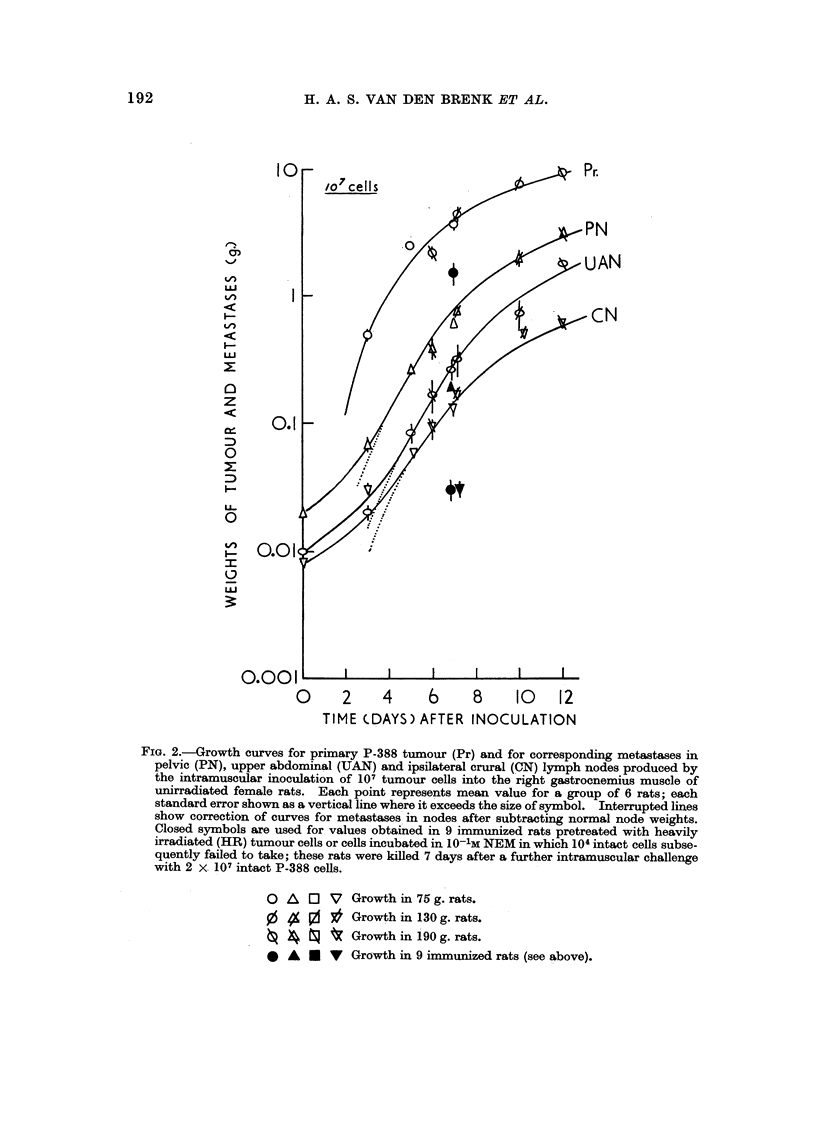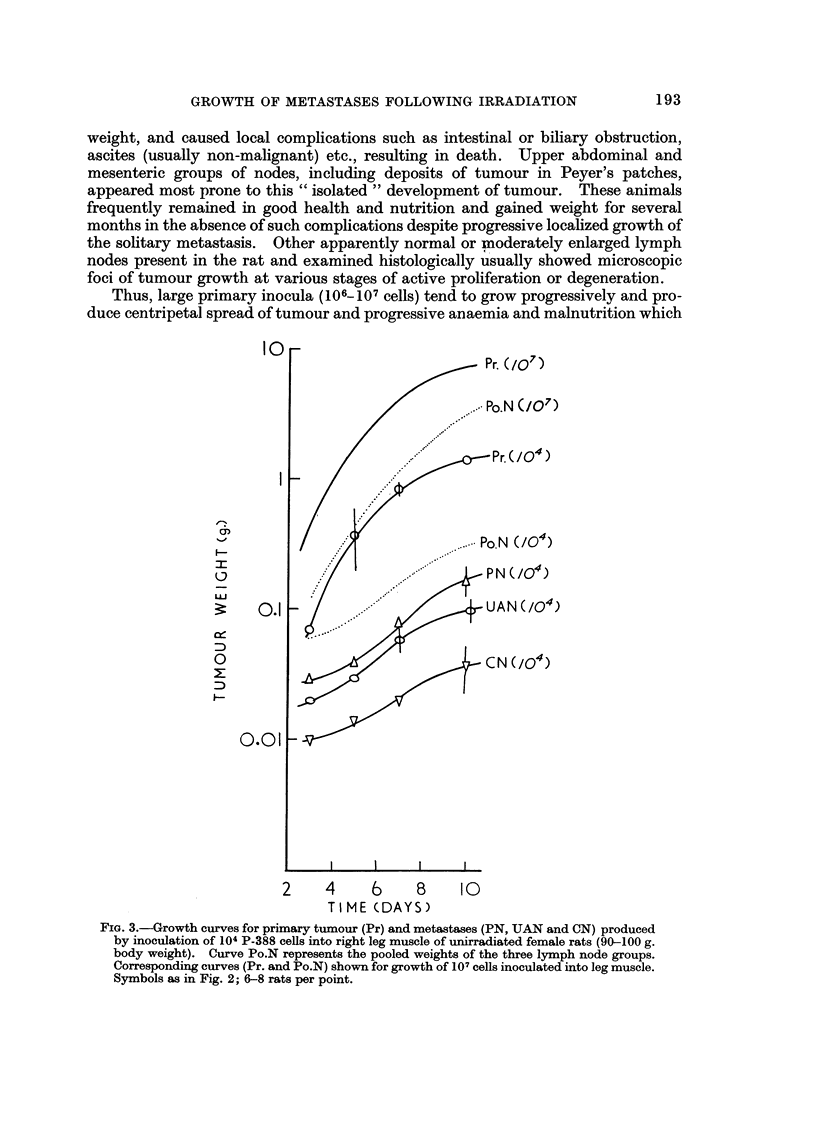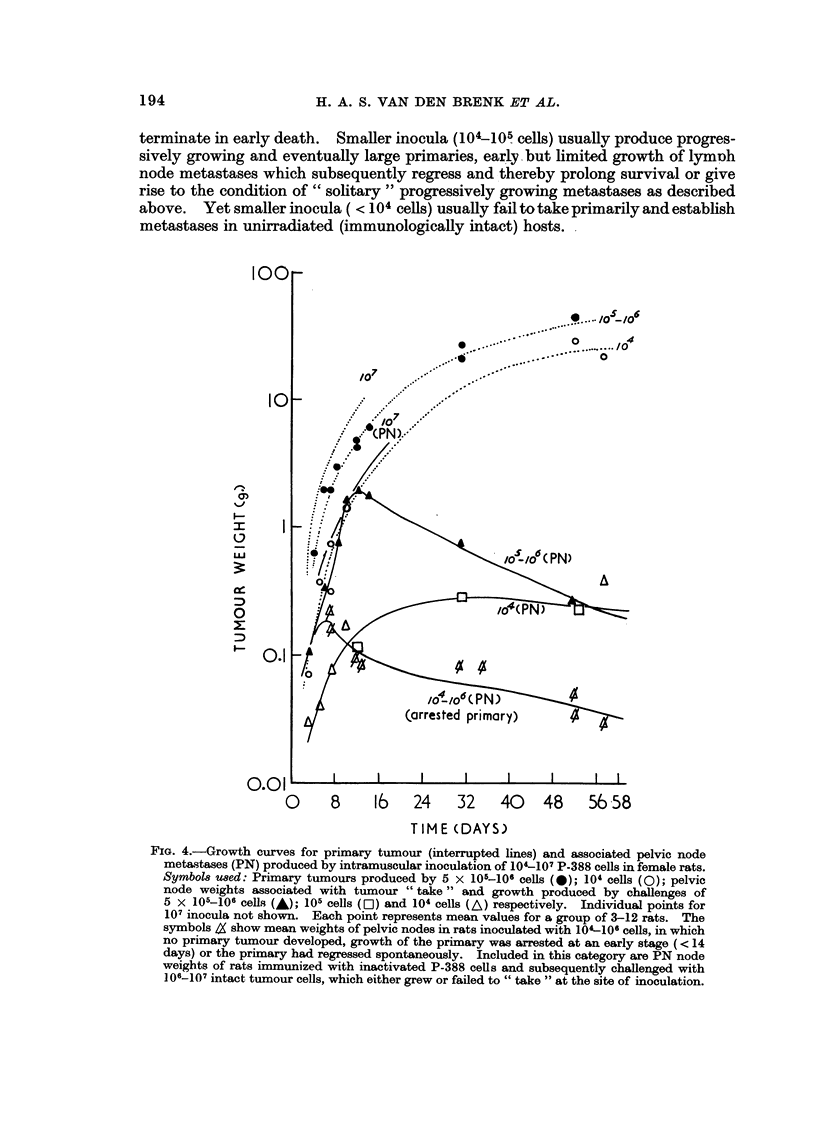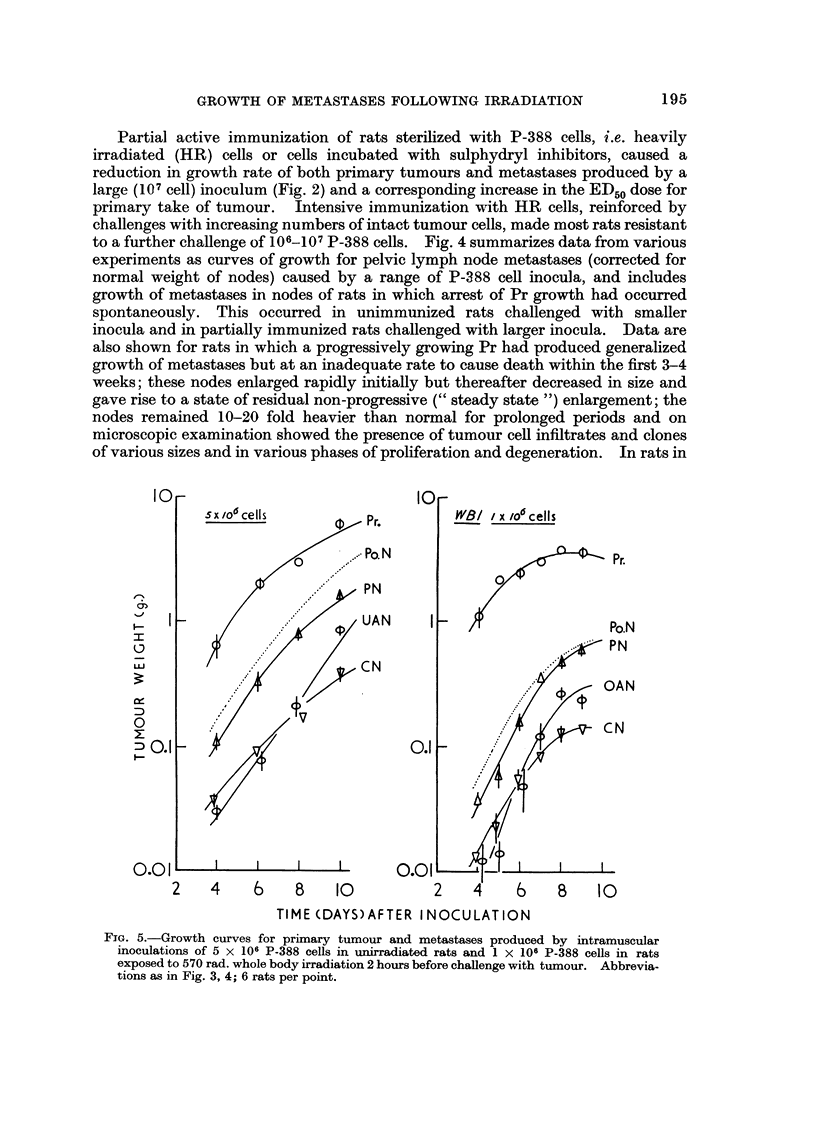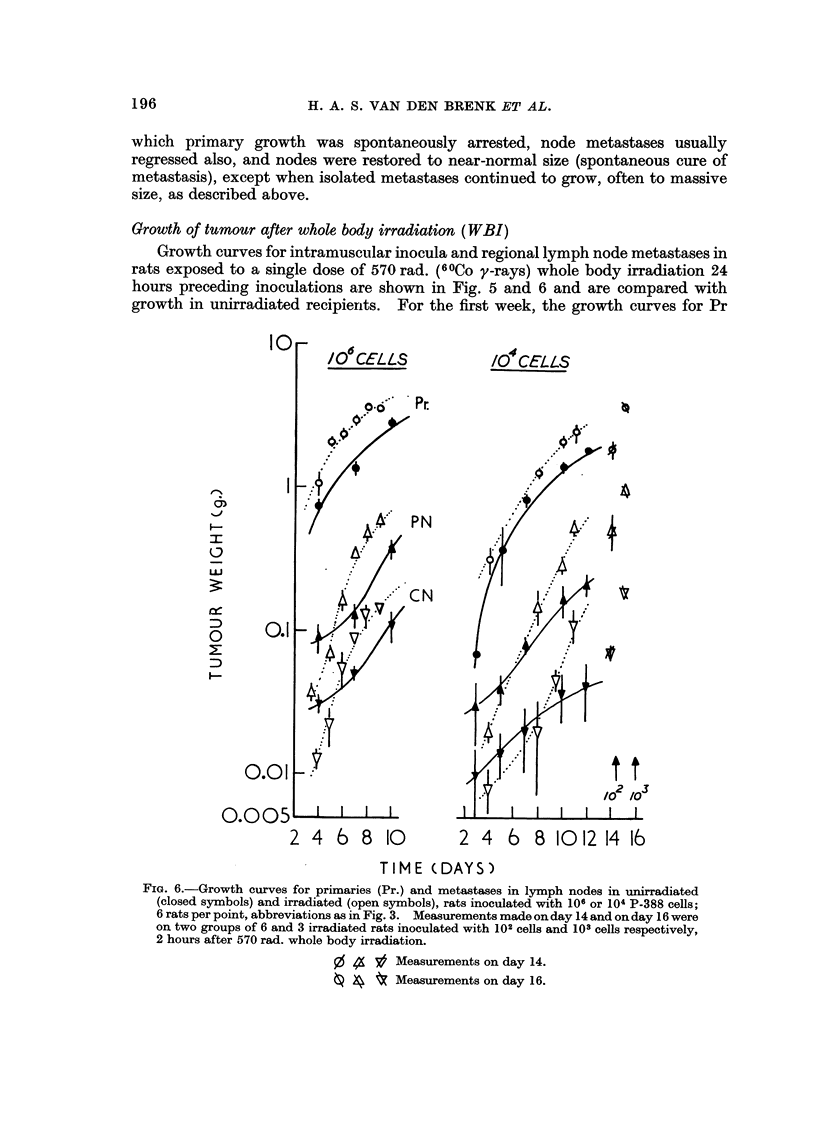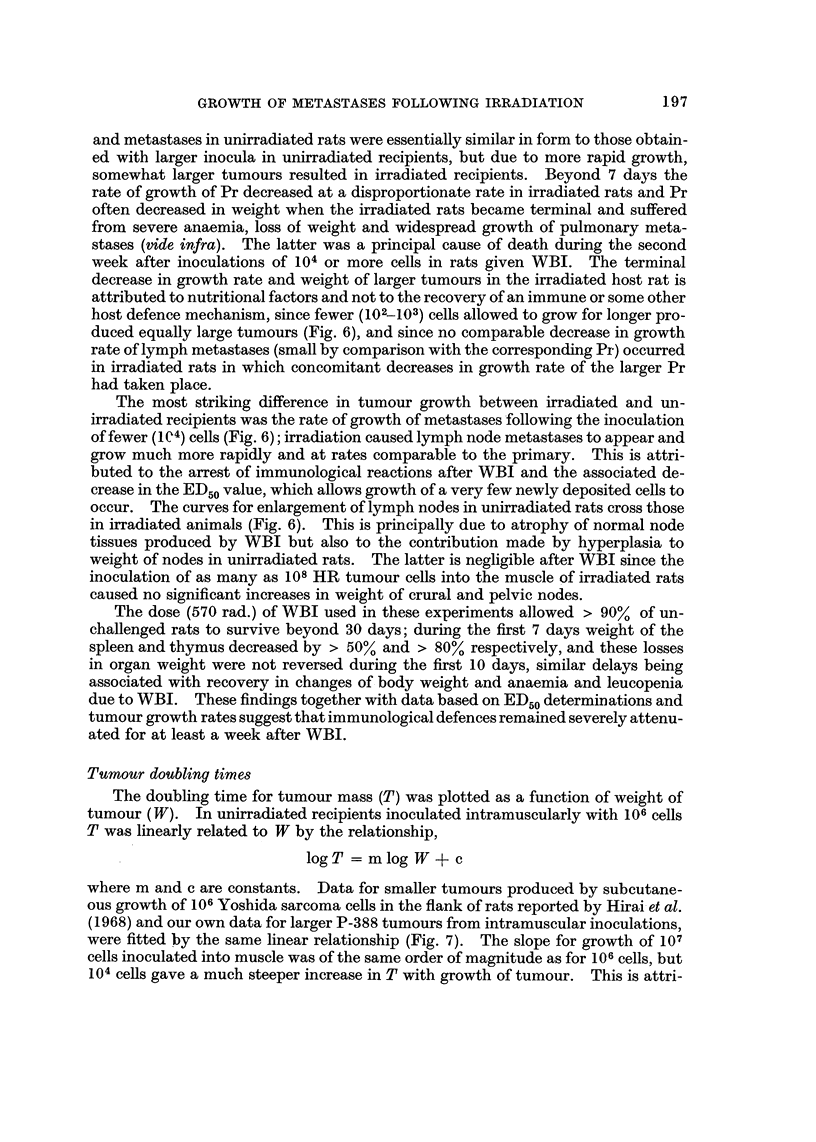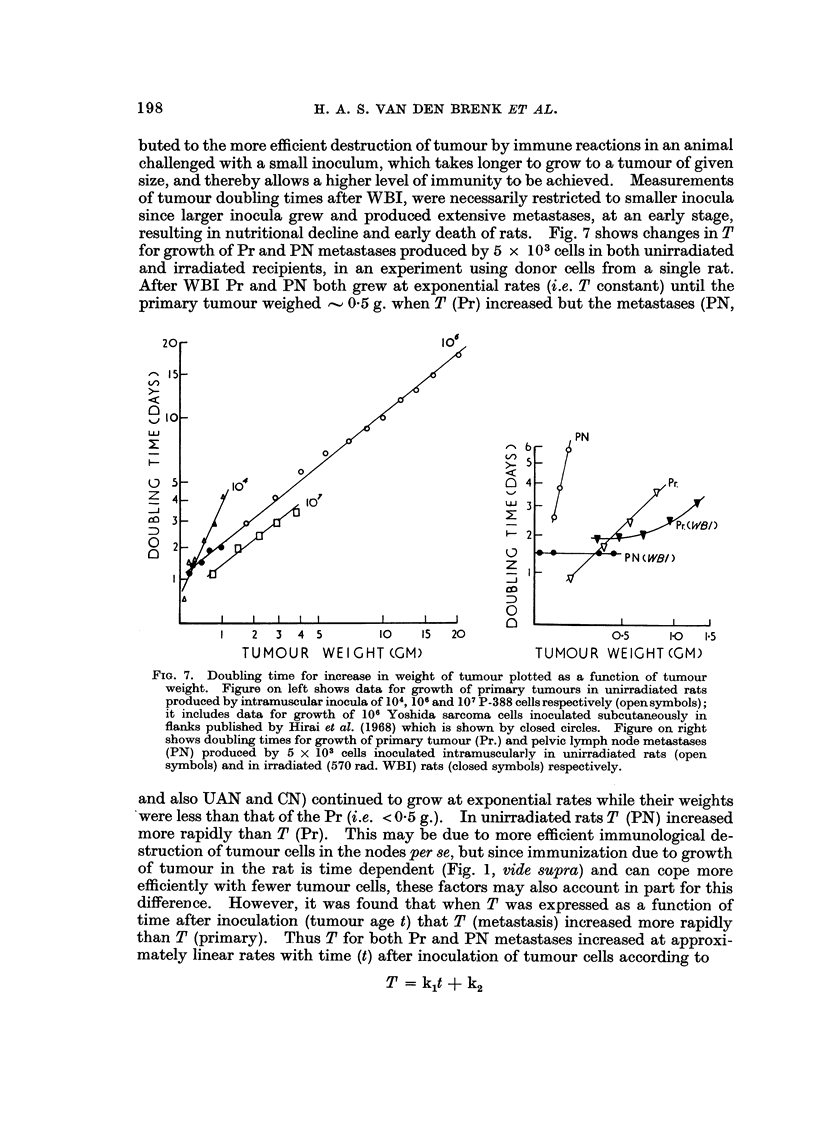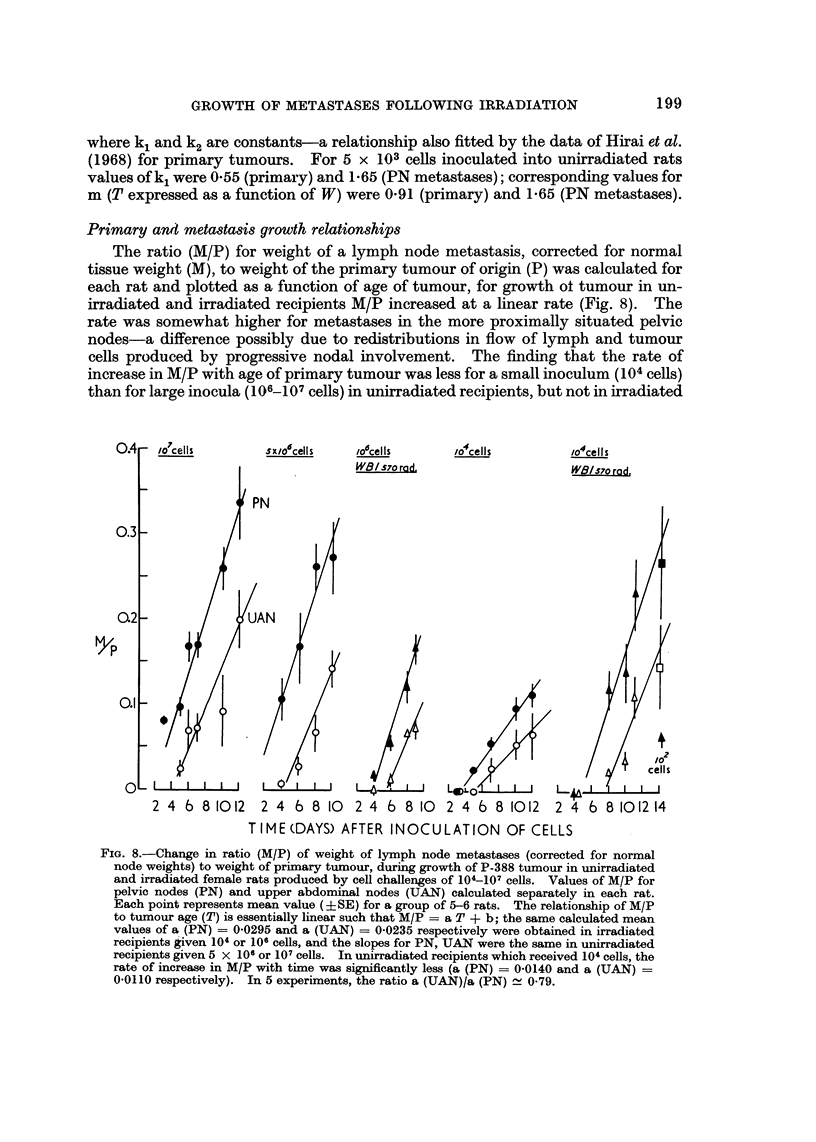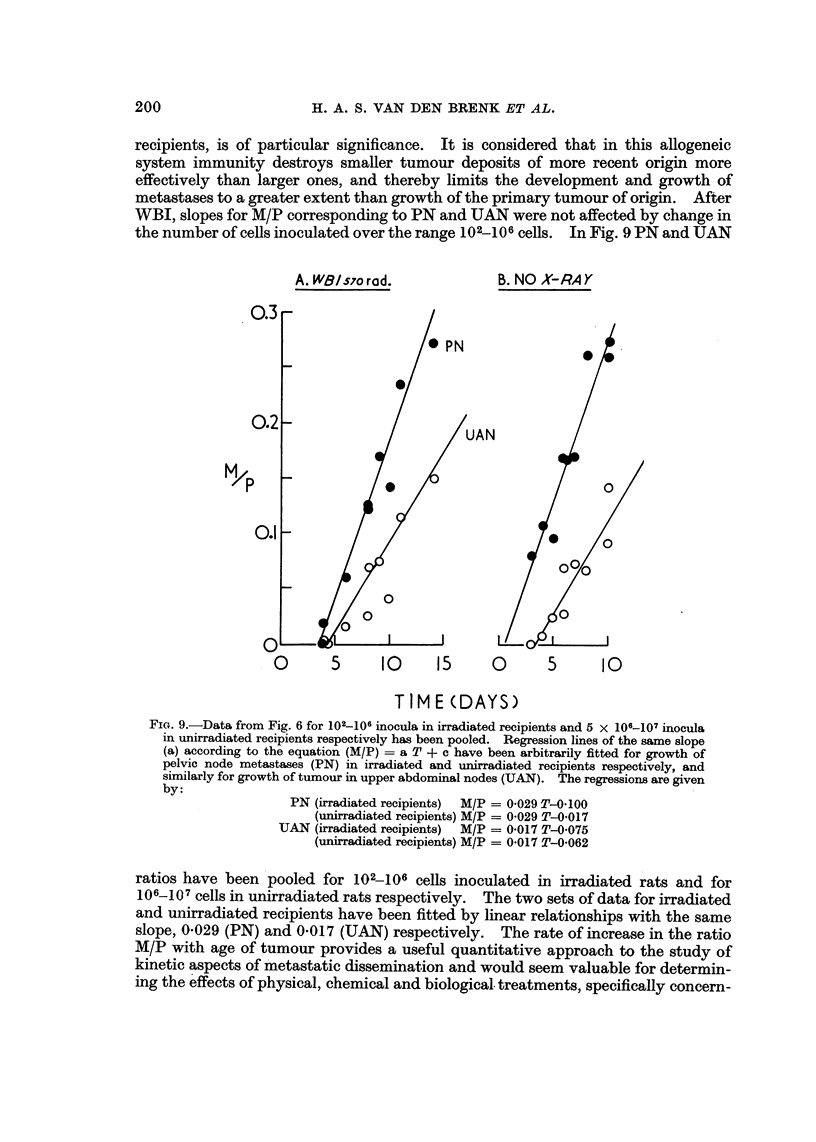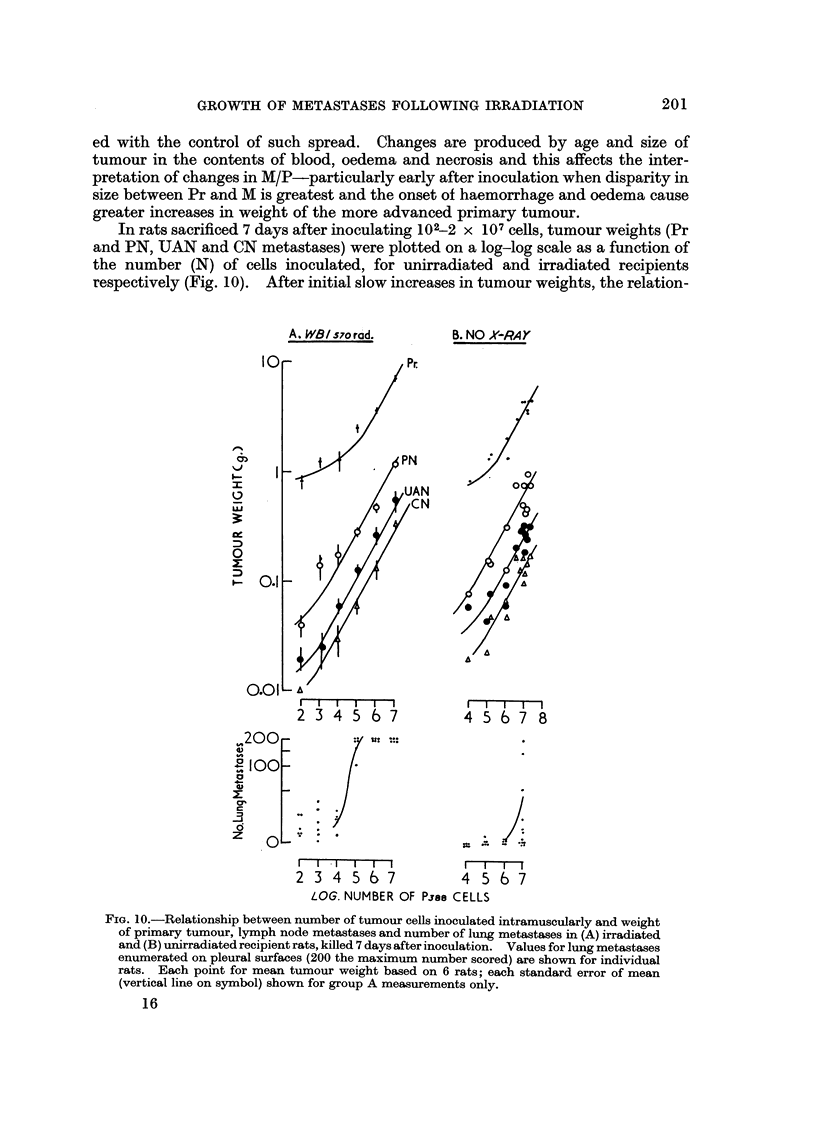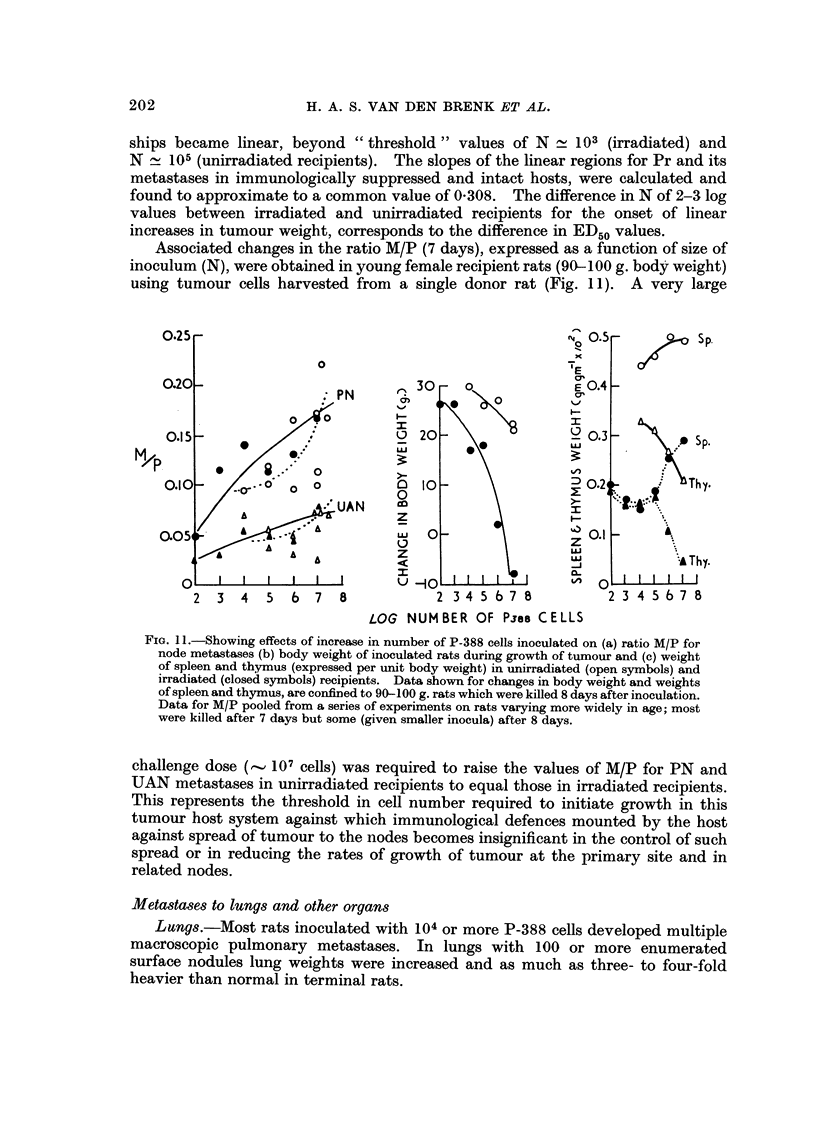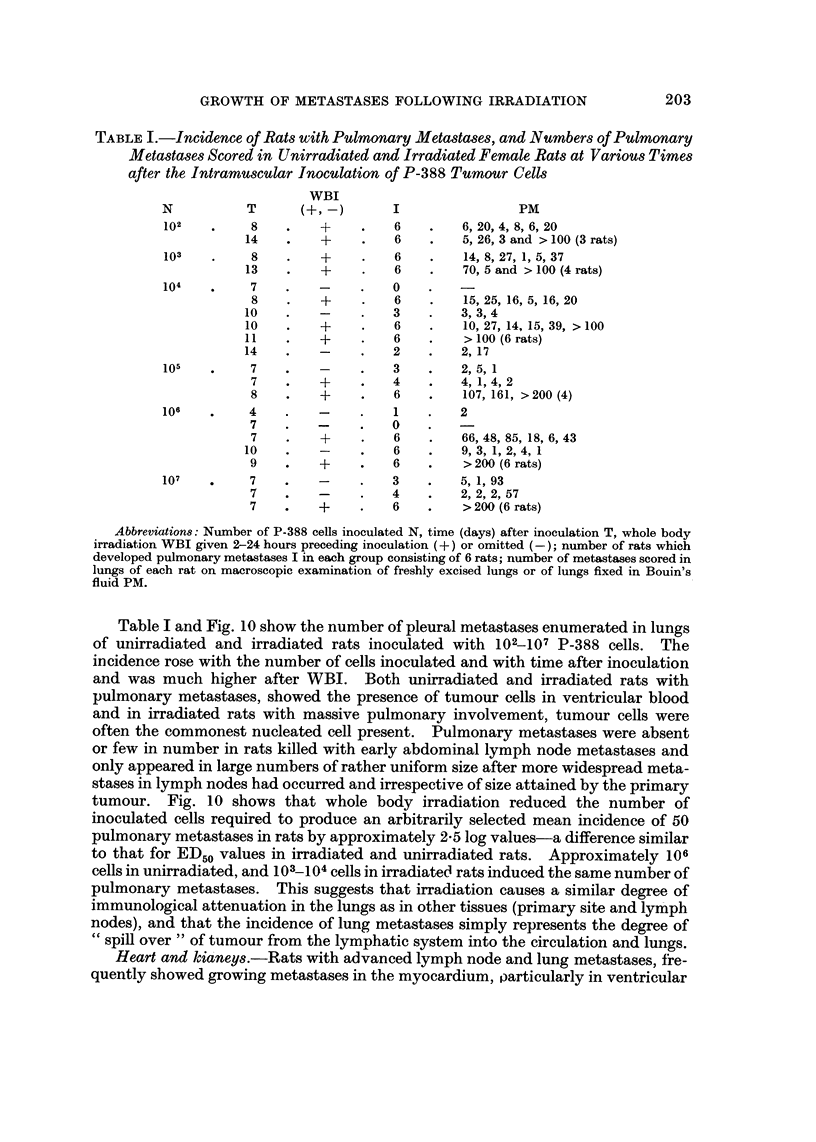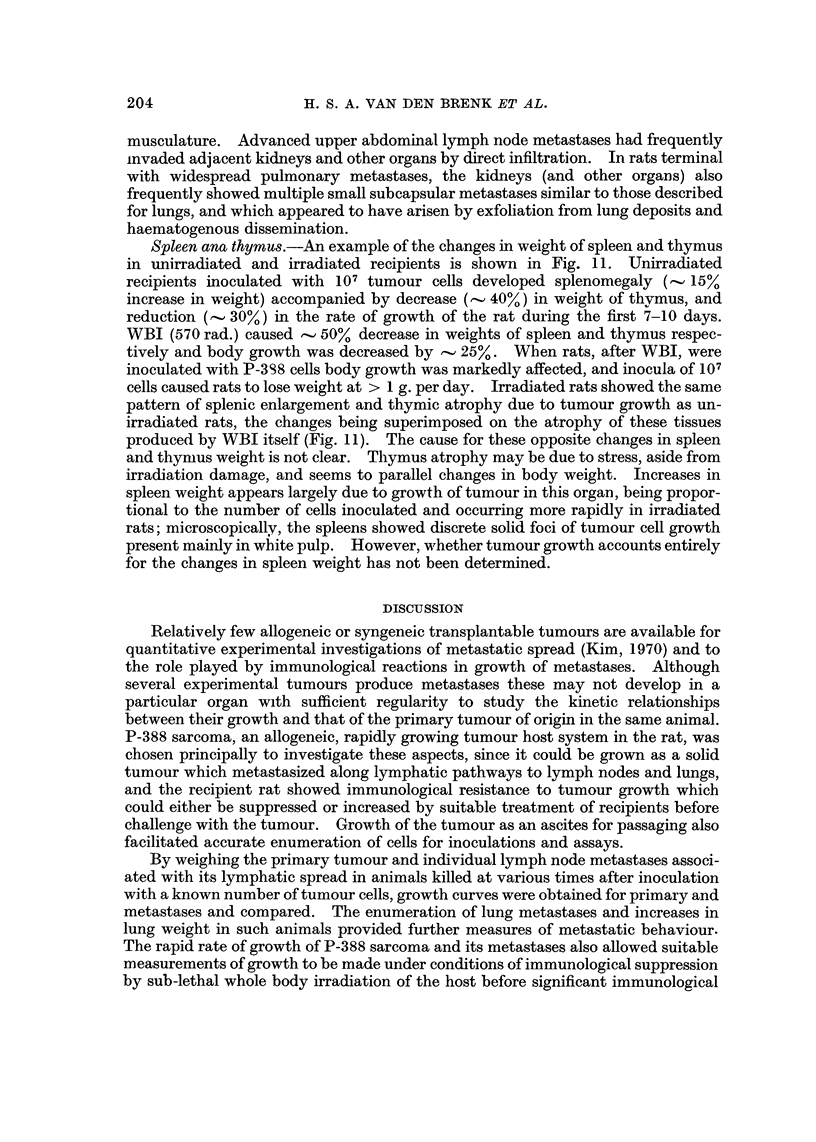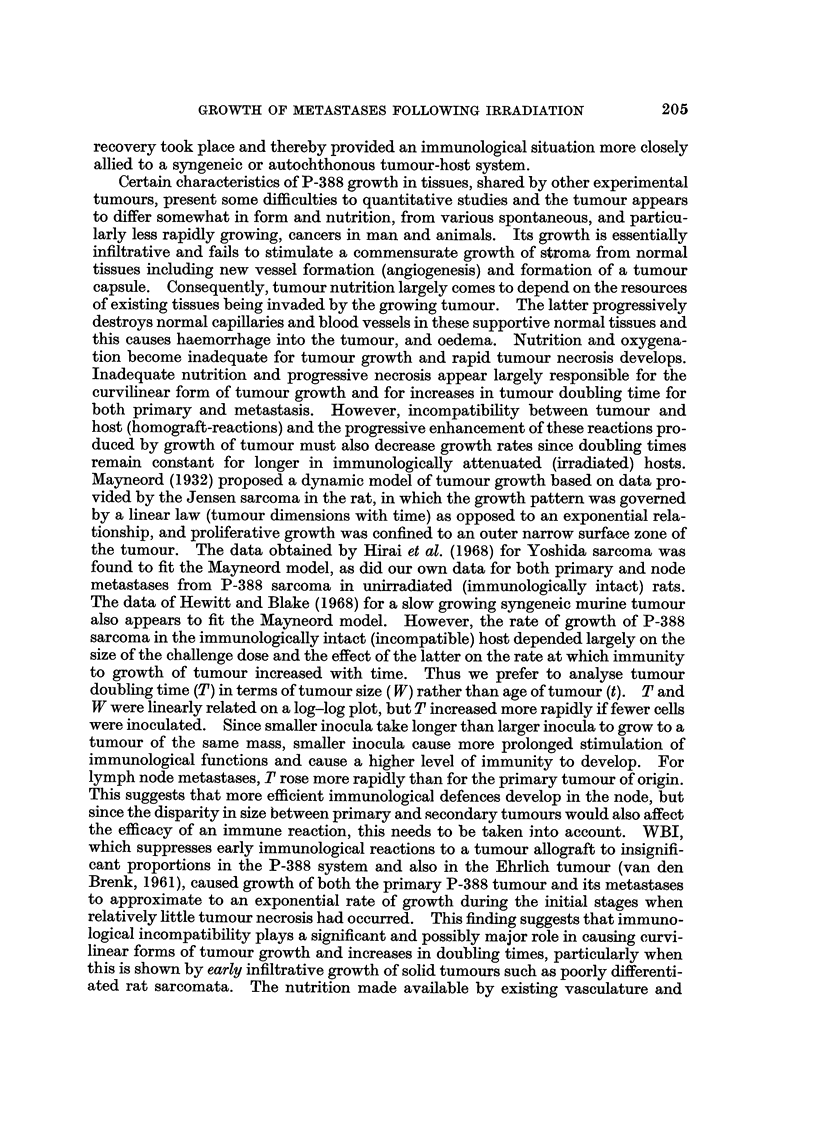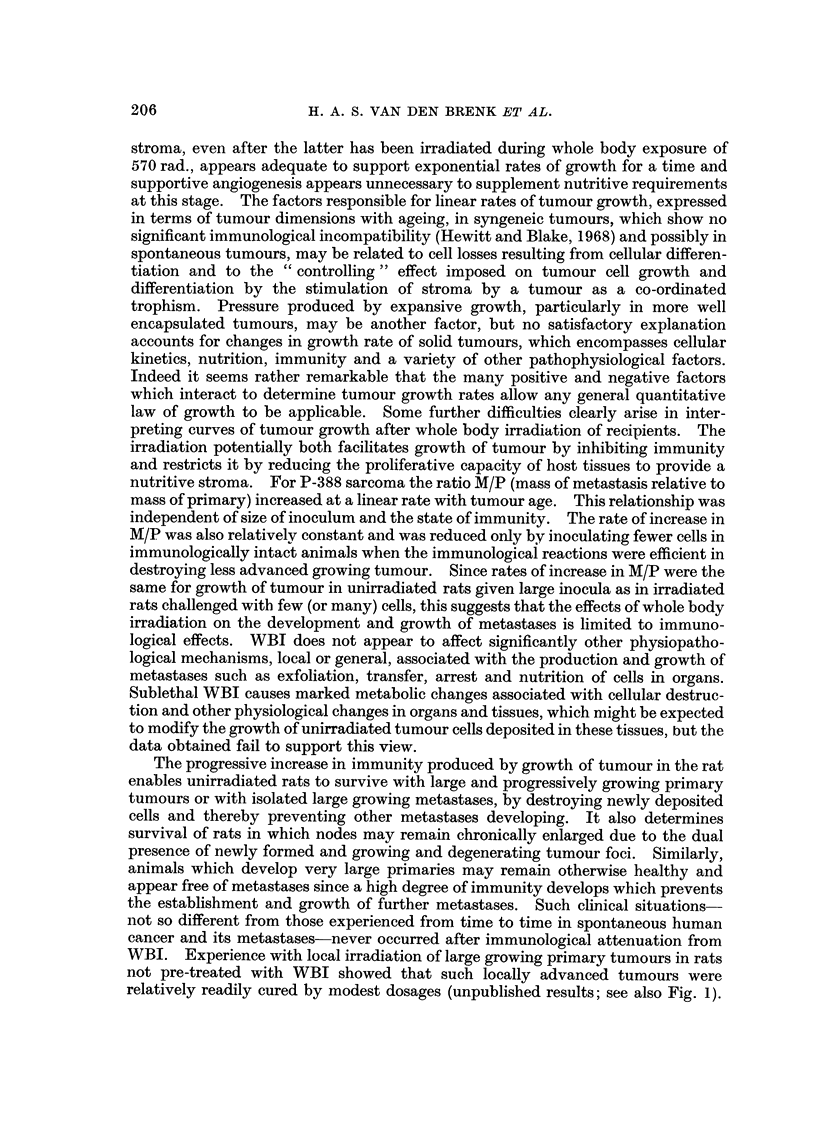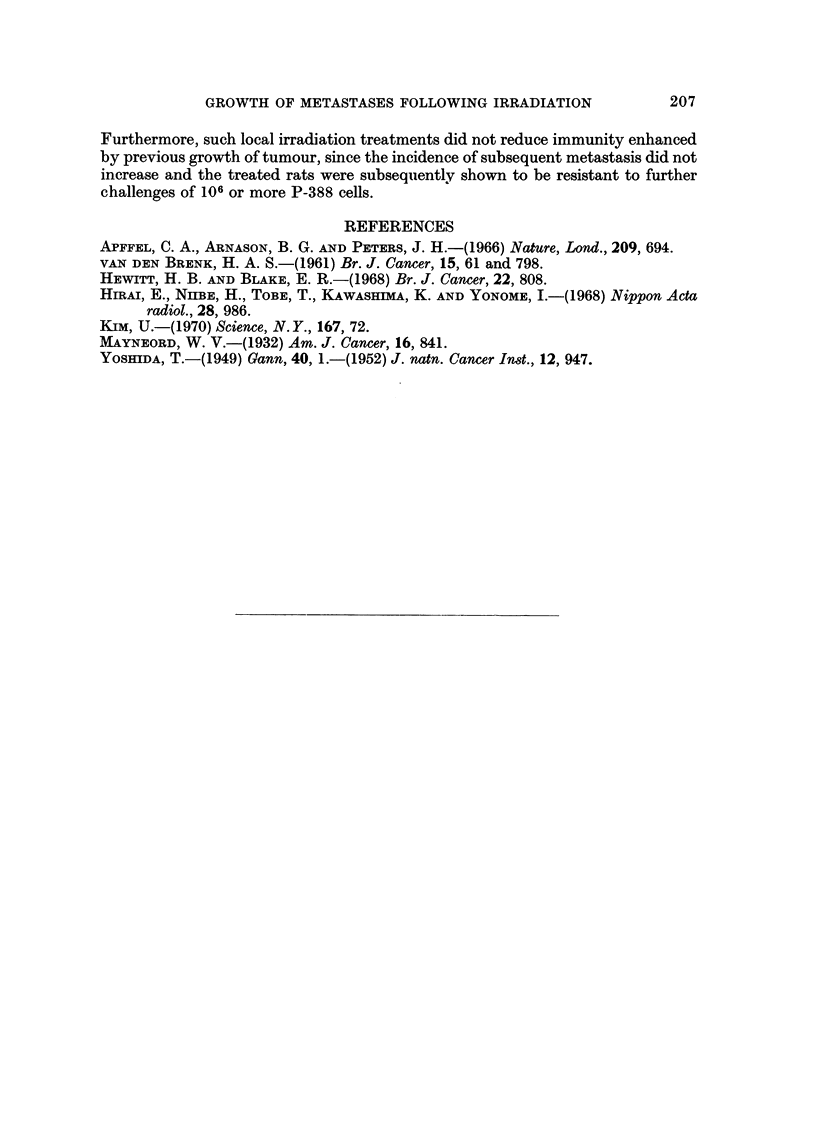# Growth of Metastases from P-388 Sarcoma in the Rat Following Whole Body Irradiation

**DOI:** 10.1038/bjc.1971.26

**Published:** 1971-03

**Authors:** H. A. S. van den Brenk, Valerie Moore, Catherine Sharpington

## Abstract

The growth of an allogeneic rapidly growing and metastasizing sarcoma (P-388) in the rat is described. Quantitative and kinetic data are provided concerning the growth of individual metastases produced in three principal regional lymph node drainage groups, and are compared with growth of the primary tumour of origin in muscle; the incidence of pulmonary metastases is also given. The effects on growth of metastases and primaries produced by sublethal whole body irradiation (WBI) before inoculations of 10-10^8^ tumour cells are described.

Growth of P-388 sarcoma in unirradiated recipients (ED_50_ ≃ 5 × 10^3^ cells) obeyed the linear growth law proposed by the Mayneord model for tumour growth, but in irradiated recipients (ED_50_ < 10 cells) early growth of primaries and metastases approximated more closely to an exponential rate of growth.

The ratio M/P of weight of metastases (M) to weight of primary tumour of origin (P) increased at a linear rate with age (t) of tumour, and gave the same slope namely, 0·029 for pelvic node metastases) in unirradiated rats inoculated with a large (10^6^-10^7^) number of cells as in irradiated rats. The slope was decreased to 0·014 for pelvic node metastases in unirradiated rats challenged with fewer (10^4^) cells but not in irradiated recipients. It is postulated that the effects of WBI on growth of metastases are confined to causing a suppression of immunity, and that WBI does not affect tumour spread significantly through other mechanisms.

In immunologically competent rats the phenomena of sequestrated progressive growth of isolated metastases in lymph nodes and of a chronic nonprogressive enlargement of nodes in which persistent tumour growth and destruction occurred side by side are described, and effects on prognoses and clinical behaviour, are discussed.

The P-388 tumour is considered of value in quantitative and kinetic experimental studies of metastases, and particularly those in which the role of immunity needs to be assessed and measured to avoid confusion with other factors or agents which might affect the spread and growth of metastases.


### GROWTH OF METASTASES FROM P-388 SARCOMA IN THE RAT FOLLOWING WHOLE BODY IRRADIATION

H. A. S. VAN DEN BRENK, VALERIE MOORE AND CATHERINE SHARPINGTON

*From the Dimbleby Research Laboratory, St. Thomas' Hospital, London, S.E.*1

Received for publication November 18, 1970

**SUMMARY.—The growth of an allogeneic rapidly growing and metastasizing sarcoma (P-388) in the rat is described. Quantitative and kinetic data are provided concerning the growth of individual metastases produced in three principal regional lymph node drainage groups, and are compared with growth of the primary tumour of origin in muscle; the incidence of pulmonary metastases is also given. The effects on growth of metastases and primaries produced by sublethal whole body irradiation (WBI) before inoculations of 10–$10^8$ tumour cells are described.**

**Growth of P-388 sarcoma in unirradiated recipients ($ED_{50} \simeq 5 \times 10^3$ cells) obeyed the linear growth law proposed by the Mayneord model for tumour growth, but in irradiated recipients ($ED_{50} < 10$ cells) early growth of primaries and metastases approximated more closely to an exponential rate of growth.**

**The ratio M/P of weight of metastases (M) to weight of primary tumour of origin (P) increased at a linear rate with age (t) of tumour, and gave the same slope namely, 0·029 for pelvic node metastases) in unirradiated rats inoculated with a large ($10^6$–$10^7$) number of cells as in irradiated rats. The slope was decreased to 0·014 for pelvic node metastases in unirradiated rats challenged with fewer ($10^4$) cells but not in irradiated recipients. It is postulated that the effects of WBI on growth of metastases are confined to causing a suppression of immunity, and that WBI does not affect tumour spread significantly through other mechanisms.**

**In immunologically competent rats the phenomena of sequestrated progressive growth of isolated metastases in lymph nodes and of a chronic nonprogressive enlargement of nodes in which persistent tumour growth and destruction occurred side by side are described, and effects on prognoses and clinical behaviour, are discussed.**

**The P-388 tumour is considered of value in quantitative and kinetic experimental studies of metastases, and particularly those in which the role of immunity needs to be assessed and measured to avoid confusion with other factors or agents which might affect the spread and growth of metastases.**

RELATIVELY few transplantable solid tumours in animals metastasize regularly from the primary site of inoculation and allow comparative and kinetic studies of growth of metastases to be made. This paper documents quantitative data concerning the rates of growth of solid metastases in lymph nodes and lungs from a rapidly growing allogeneic sarcoma P-388 in the rat, and compares growth in unirradiated (immunologically intact) recipients with growth in rats given sublethal whole body irradiation before tumour inoculations to suppress the immunological reactions to tumour growth.

### MATERIALS AND METHODS

### *Tumour*

The P-388 sarcoma used in these experiments was a nitrogen-mustard resistant strain of Yoshida ascites sarcoma, which was kindly provided by Dr. T. A. Connors, Chester Beatty Institute, London.

The original Yoshida sarcoma could be grown either as an ascitic or solid tumour and was considered to be of reticulo-endothelial derivation (Yoshida, 1949, 1952). The P-388 variant behaves similarly and resembles an anaplastic reticulosis in its mode of growth and spread. The tumour metastasizes freely and rapidly along associated lymphatic pathways to form metastatic deposits in regional lymph nodes. At a later stage centripetal lymphatic spread produces more widespread node metastases. Tumour cells which enter the main lymphatic ducts are carried into the venous circulation, traverse the right heart and arrest in the lungs to produce discrete pulmonary metastases. Larger solid deposits of tumour—both at the primary site and metastases—are characterized by an outer narrow rapidly proliferating and invasive zone, an intermediate intensely haemorrhagic zone consisting of both viable and necrotic foci of tumour separated by lakes of stagnant blood and haemorrhage, and a central zone of necrotic tumour which predominates in larger older tumours. Thus in structure the P-388 tumour conforms with the model system of tumour growth proposed by Mayneord (1932). Histological and micro-angiographic studies have shown that this tumour fails to stimulate significant growth of new blood vessels (angiogenesis) and other tissues which constitute a vascular stroma required to support its continued growth and to prevent necrosis (to be published); viable proliferating tumour primarily utilizes the capillary beds of the invaded tissues for its nutrition and metabolism. Intratumoral haemorrhage causes growing solid deposits to be blood-red in colour and this facilitates the enumeration of pulmonary metastases in freshly excised lungs for quantitative purposes.

### *Passage and inoculation*

The P-388 tumour was passaged every 4–7 days in female Caworth Farm (Wistar) rats of a specific pathogen free strain by intraperitoneal inoculation of 1–2 × $10^6$ P-388 cells. Suitably diluted tumour ascites fluid was counted in a Coulter (model A) electronic cell counter. A parallel differential count of tumour cells and erythrocytes was made on each donor sample to correct for contamination with blood. In samples of 4–5-day-old ascites fluid used for inoculation, $< 5\%$ of erythrocytes and $< 1\%$ of leucocytes and macrophages were usually present and tests for cell viability based on a dye-exclusion technique with 0·1% nigrosin showed that $< 0{\cdot}1\%$ tumour cells appeared non-viable.

P-388 ascites fluid was diluted with ice cold Tyrode's buffer (pH 7·6), and 0·1 ml. containing the required number of cells was inoculated intramuscularly into the distal third of the gastrocnemius muscle above the ankle joint of the rat. A group of 6–8 rats were used for each point in constructing tumour growth curves.

Tumour inoculations made into the leg muscle of rats gave rise to diffuse thickenings which expanded the leg into a pyramidal shape. The using of calipers to determine mean dimensions was found both tedious and inaccurate. Consequently growth of tumour in the leg was scored at 1–2 day intervals in individual rats which were randomized, using a semi-quantitative index of the size (0–6) graded as: no tumour palpable (0); slight thickening (1); spindle shaped thickening of distal $\frac{1}{3}$–$\frac{1}{2}$ of leg muscle but no visible deformity (2); whole calf thickened, with visible enlargement and encroaching on popliteal fossa (3); large triangular shaped tumefaction of whole calf and thigh muscle causing flexion deformity (4); and whole hindquarter involved by large tumour with oedema of foot (6).

To weigh primary tumour and metastases, the rat was killed by an overdose of pentobarbital Na and dissected. The ipsilateral (inoculated side) and contralateral popliteal nodes were removed through longitudinal incisions over each fossa and weighed. Then both hind limbs were amputated at the same level and weighed separately to determine weight of the primary tumour by subtraction. To calculate the error involved in excising and weighing a limb, the right hind limb was excised in groups of six normal rats, weighing 100–120 g., and the mean weight of limb per unit body weight determined. The latter was found to be associated with a standard deviation of $\pm$ 5%, and it was calculated that a difference in weight of not less than 0·6 g. between the intact and tumour-bearing legs was required to be significant at the 0·05 level. Next, the abdomino-thoracic skin was incised longitudinally, widely elevated and retracted to expose inguinal, axillary and submandibular nodes on both sides. Any macroscopic evidence of metastases was recorded, and in some experiments the inguinal nodes were removed and weighed. The abdomen was opened, and the vagina and rectum divided as low as possible; the uterus and adnexa, and rectum were stripped to expose the central groups of lower and upper abdominal nodes which were removed and weighed. The lower abdominal nodes (referred to as the " pelvic " group) were defined as all nodes contained within the bifurcation of the aorta (presacral and iliac nodes) together with all paraortic nodes distal to the renal vessels, but excluding nodes contained within the leaves of the mesentery and mesocolon. The " upper abdominal node " group was constituted by all retroperitoneal paraortic and retroaortic nodes situated between the origin of the renal vessels and the crura of the diaphragm, *i.e.* the nodes related to the coeliac axis and the origin of the thoracic duct, including the retroaortic node which is partly hidden by the left crus of the diaphragm. Lymphatic dissemination of the tumour from the primary inoculation site in the leg involved early spread to ipsilateral popliteal (crural (CN)) and pelvic nodes, followed by subsequent spread to upper abdominal nodes, inguinal and mesenteric nodes. Once upper abdominal node metastases were present, and often earlier, a more widespread dissemination of tumour to more outlying lymph node groups (mediastinal and thymic groups, axillary and even submandibular lymph nodes) was seen. In animals with advanced tumours, and particularly after whole body irradiation, the contralateral inguinal nodes and contra-lateral popliteal nodes became macroscopically involved and enlarged. The number of metastases present on the pleural surfaces of both lungs were counted when their number did not exceed 200. In lungs with 200 or more pleural metastases it was not possible to make sufficiently reliable counts, and these rats were scored arbitrarily as having 200 (maximum) metastases. In most experiments the spleen and thymus were removed and weighed, and individual changes in body weight of rats were also recorded.

The weights of the primary tumour in the leg (P) ipsilateral popliteal nodes (CN), pelvic nodes (PN) and upper abdominal nodes (UAN), respectively were used to construct corresponding growth curves. The incidence of pulmonary metastases largely represented the " final spill-over " of tumour metastasis in the lymphatic system into the circulation. The corresponding weights of primary tumour (P) and metastasis (M) were used to analyse the rates of production of metastases and relative rates of growth by determining changes in the ratio M/P with time (T) after inoculation, or by altering the number (N) of tumour cells inoculated and weighing the tumours at the same post-inoculation time. In uninoculated rats the normal weights of pelvic, upper abdominal and crural lymph nodes were $<0 \cdot 04$, $<0 \cdot 03$, and $<0 \cdot 01$ g. respectively in unirradiated rats and $<0 \cdot 02$, $<0 \cdot 01$ and $<0 \cdot 005$ g. respectively after 570 rad. WBI. To calculate M/P ratios, the maximum weights of normal lymph nodes were subtracted from weights of corresponding involved nodes in the inoculated rat to obtain more accurate values for weight of tumour metastases. In unirradiated rats reactive enlargement of nodes must also be taken into account. It was found that the injection on three successive days of $1\text{–}5 \times 10^7$ P-388 cells subjected to a high dose (4–6 krad.) X-radiation *in vitro* to prevent their growth (HR cells) did not cause the regional nodes to increase to more than double their normal weight. Nodes weighing more than 0·08 (PN), 0·06 (UAN) and 0·02 (CN) g. respectively in unirradiated rats, or more than 0·02 (PN), 0·01 (UAN) and 0·01 (CN) g. in irradiated recipients invariably showed macroscopic and microscopic evidence of tumour growth, and these values were taken to represent the upper limits of normality even in the presence of reactive (hyperplastic) changes.

*Irradiation techniques*

A twin-headed therapeutic Cobalt 60 unit, kindly made available by Dr. I. Churchill-Davidson, was used to irradiate uniformly a box containing 10–12 rats placed midway between the two sources. The mean dose rate in tissue from the two sources (totalling 8273 Ci) was 157 rad. $min^{-1}$. The rats received a single whole body exposure of 570 rad. to suppress immunological functions, usually 1–4 hours before inoculation of P-388 cells, but in some experiments 4–16 hours elapsed between irradiation and inoculation. Twin opposed X-ray beams operated at 250 kv 15 mA with added filtration to give a HVL 1·0 mm. Cu were used to irradiate tumour cells *in vitro* and also to locally irradiate the limb tumour, the body of the anaesthetized animal being shielded between 2 mm. thick lead plates containing appropriate cutouts to expose the tumour bearing limb tissues only.

*Tumour " take " and $ED_{50}$*

The number of P-388 cells required to produce palpable (Grade 1–2) tumours at the site of primary inoculation in muscle within 4 weeks after inoculation in 50% of rats was taken to represent the $ED_{50}$ dose.

*Double leg inoculations*

In certain experiments the host's immunological capacity to react to P-388 cells was assessed in terms of the growth of a second intramuscular challenge of the tumour to the contralateral leg (termed *second* challenge), given at intervals after the first challenge.

*Immunization of rats against P-388 cells*

In some experiments, active immunization involved the use of P-388 cells sterilized by heavy irradiation (HR) or by incubation at 37° C. for 30 minutes with a sulphydryl inhibitor, sodium iodoacetate (NaIOA) or N-ethylmaleimide (NEM) as described by Apffel *et al.* (1966). It was found that the P-388 tumour cells were unusually resistant to killing by these reagents; incubation in concentrations of $10^{-2}$ M NaIOA and $10^{-1}$ M NEM respectively were required to prevent growth of $10^{6}$–$10^{7}$ tumour cells in rats which had *not* received whole body irradiation. It was also found that P-388 cells stored frozen at — 70° C. in the presence of glycerol or dimethylsulphoxide, and irradiated with 4000–6000 rads after thawing, were much less effective for immunizing of rats than freshly removed and irradiated cells; NaIOA also proved more efficacious than NEM in this respect. Consequently, to increase immunity irradiated non-frozen freshly removed cells, or freshly removed cells incubated with $10^{-2}$ M NaIOA or $10^{-1}$ M NEM, were used. Rats received 1–4 × $10^{7}$ inactivated P-388 cells subcutaneously twice weekly for 3 weeks, and were challenged intramuscularly a week later with $10^{5}$ intact P-388 cells. If the latter failed to " take " and grow, the rats were regarded as hyperimmune and a further $10^{6}$–$10^{7}$ viable cells were inoculated and their growth compared with the growth in unimmunized rats.

### RESULTS

*$ED_{50}$ values (tumour take)*

The $ED_{50}$ value for intramuscular inoculations was approximately 5 × $10^{3}$ tumour cells in unirradiated recipients and < 10 cells in rats pre-irradiated with 570 rad. WBI. Repeated assays performed over the preceding year gave $ED_{50}$ values not significantly different from these values. In unirradiated recipients $10^{2}$ cells rarely " took " to produce palpable tumour or metastases; $10^{5}$ cells invariably produced local growth of tumour, but metastases often failed to show up macroscopically, whilst $10^{6}$–$10^{7}$ cells produced metastases which grew and killed most rats within 3–6 weeks.

For subcutaneous inoculations made into the tissues of the neck overlying the salivary glands, $ED_{50}$ values were similar to those obtained for the intramuscular route, but values were higher for subcutaneous inoculations into the foot and tail.

*Immunization produced by growth of tumour*

In rats challenged with the tumour in one leg, which was either allowed to grow for varying times or treated with a *local* single dose of 1600 rad. X-rays to arrest its growth, a second challenge with $10^{6}$ P-388 cells into the contralateral leg grew less well depending on the interval elapsing between challenges. Fig. 1 shows growth of second challenges ($10^{6}$ cells) in 72 rats given 0–50 days after a primary challenge with $10^{7}$ cells. A progressive increase in immunity occurred during growth of the first challenge, even if tumour growth was prevented or reduced by local irradiation administered before the second challenge was given. Fifty days after rats were challenged with $10^{7}$ cells, and the resulting 7–14-day-old tumours had been irradiated to inhibit their growth so as to allow the rats to survive, a second challenge of $10^{6}$ cells failed to take and grow in the opposite leg.

*Curves of tumour growth (unirradiated hosts)*

Growth curves for the primary leg tumour (Pr) at site of intramuscular inoculation and for lymph node metastases (PN, UAN, CN) in unirradiated female rats inoculated with $10^{7}$ or $10^{4}$ P-388 cells are shown in Fig. 2 and 3 respectively. Similar curvilinear, relationships were obtained for Pr and its metastases in lymph nodes. Tumour growth rates characteristically decreased as tumours enlarged, *i.e.* the doubling time ($T$) for tumour mass increased (*vide infra*). Initially weight of Pr increased very rapidly ($T < 24$ hours) as did weight of lymph nodes infiltrated with tumour in rats given a large inoculum ($10^7$ cells). A smaller inoculum ($10^4$ cells) produced a more rapid decline in growth rate of Pr than larger inocula and an even more marked reduction in the rate of growth of lymph node metastases. The

FIG. 1.—Growth curves for second challenges of $10^6$ P-388 cells inoculated into the contralateral leg of female rats (solid lines) at various times after the inoculation of $10^7$ cells in the ipsilateral leg. Growth curves for single (first) challenges with $10^5$ P-388 cells in unirradiated rats (×———×) and in rats 2 hours after wholebody irradiation (× - - - - ×) are shown for comparative purposes. Measurement of size of tumour has been based on the index (Grades 0–6), as described under Methods. Data is for ten groups, each composed of 6–9 rats.

enlarged lymph nodes produced by smaller inocula showed early microscopic evidence of tumour cell proliferation with the presence of tumour cell clones, but these metastases usually failed to progress and survive; 2–3 weeks after inoculation the popliteal and abdominal nodes in most rats remained either moderately enlarged or had regressed, despite further growth of primary (*vide infra*). However, in a proportion of animals tumour continued to grow in a single node or node group after growth in other nodes had ceased and often completely regressed. In such animals a large solitary tumour metastasis had not infrequently grown to 50 g. or more in

FIG. 2.—Growth curves for primary P-388 tumour (Pr) and for corresponding metastases in pelvic (PN), upper abdominal (UAN) and ipsilateral crural (CN) lymph nodes produced by the intramuscular inoculation of $10^7$ tumour cells into the right gastrocnemius muscle of unirradiated female rats. Each point represents mean value for a group of 6 rats; each standard error shown as a vertical line where it exceeds the size of symbol. Interrupted lines show correction of curves for metastases in nodes after subtracting normal node weights. Closed symbols are used for values obtained in 9 immunized rats pretreated with heavily irradiated (HR) tumour cells or cells incubated in $10^{-1}$M NEM in which $10^4$ intact cells subsequently failed to take; these rats were killed 7 days after a further intramuscular challenge with $2 \times 10^7$ intact P-388 cells.

○ △ □ ▽ Growth in 75 g. rats.
Ø ⍋ ⊠ ⍒ Growth in 130 g. rats.
⦸ ⍋ ⊠ ⍒ Growth in 190 g. rats.
● ▲ ■ ▼ Growth in 9 immunized rats (see above).

weight, and caused local complications such as intestinal or biliary obstruction, ascites (usually non-malignant) etc., resulting in death. Upper abdominal and mesenteric groups of nodes, including deposits of tumour in Peyer's patches, appeared most prone to this " isolated " development of tumour. These animals frequently remained in good health and nutrition and gained weight for several months in the absence of such complications despite progressive localized growth of the solitary metastasis. Other apparently normal or moderately enlarged lymph nodes present in the rat and examined histologically usually showed microscopic foci of tumour growth at various stages of active proliferation or degeneration.

Thus, large primary inocula ($10^6$–$10^7$ cells) tend to grow progressively and produce centripetal spread of tumour and progressive anaemia and malnutrition which

FIG. 3.—Growth curves for primary tumour (Pr) and metastases (PN, UAN and CN) produced by inoculation of $10^4$ P-388 cells into right leg muscle of unirradiated female rats (90–100 g. body weight). Curve Po.N represents the pooled weights of the three lymph node groups. Corresponding curves (Pr. and Po.N) shown for growth of $10^7$ cells inoculated into leg muscle. Symbols as in Fig. 2; 6–8 rats per point.

terminate in early death. Smaller inocula ($10^4$–$10^5$ cells) usually produce progressively growing and eventually large primaries, early but limited growth of lymph node metastases which subsequently regress and thereby prolong survival or give rise to the condition of " solitary " progressively growing metastases as described above. Yet smaller inocula ( < $10^4$ cells) usually fail to take primarily and establish metastases in unirradiated (immunologically intact) hosts.

FIG. 4.—Growth curves for primary tumour (interrupted lines) and associated pelvic node metastases (PN) produced by intramuscular inoculation of $10^4$–$10^7$ P-388 cells in female rats. *Symbols used:* Primary tumours produced by 5 × $10^5$–$10^6$ cells (●); $10^4$ cells (○); pelvic node weights associated with tumour " take " and growth produced by challenges of 5 × $10^5$–$10^6$ cells (▲); $10^5$ cells (□) and $10^4$ cells (△) respectively. Individual points for $10^7$ inocula not shown. Each point represents mean values for a group of 3–12 rats. The symbols ⩓̸ show mean weights of pelvic nodes in rats inoculated with $10^4$–$10^6$ cells, in which no primary tumour developed, growth of the primary was arrested at an early stage ( < 14 days) or the primary had regressed spontaneously. Included in this category are PN node weights of rats immunized with inactivated P-388 cells and subsequently challenged with $10^6$–$10^7$ intact tumour cells, which either grew or failed to " take " at the site of inoculation.

Partial active immunization of rats sterilized with P-388 cells, *i.e.* heavily irradiated (HR) cells or cells incubated with sulphydryl inhibitors, caused a reduction in growth rate of both primary tumours and metastases produced by a large ($10^7$ cell) inoculum (Fig. 2) and a corresponding increase in the $ED_{50}$ dose for primary take of tumour. Intensive immunization with HR cells, reinforced by challenges with increasing numbers of intact tumour cells, made most rats resistant to a further challenge of $10^6$–$10^7$ P-388 cells. Fig. 4 summarizes data from various experiments as curves of growth for pelvic lymph node metastases (corrected for normal weight of nodes) caused by a range of P-388 cell inocula, and includes growth of metastases in nodes of rats in which arrest of Pr growth had occurred spontaneously. This occurred in unimmunized rats challenged with smaller inocula and in partially immunized rats challenged with larger inocula. Data are also shown for rats in which a progressively growing Pr had produced generalized growth of metastases but at an inadequate rate to cause death within the first 3–4 weeks; these nodes enlarged rapidly initially but thereafter decreased in size and gave rise to a state of residual non-progressive (" steady state ") enlargement; the nodes remained 10–20 fold heavier than normal for prolonged periods and on microscopic examination showed the presence of tumour cell infiltrates and clones of various sizes and in various phases of proliferation and degeneration. In rats in

FIG. 5.—Growth curves for primary tumour and metastases produced by intramuscular inoculations of $5 \times 10^6$ P-388 cells in unirradiated rats and $1 \times 10^6$ P-388 cells in rats exposed to 570 rad. whole body irradiation 2 hours before challenge with tumour. Abbreviations as in Fig. 3, 4; 6 rats per point.

which primary growth was spontaneously arrested, node metastases usually regressed also, and nodes were restored to near-normal size (spontaneous cure of metastasis), except when isolated metastases continued to grow, often to massive size, as described above.

*Growth of tumour after whole body irradiation (WBI)*

Growth curves for intramuscular inocula and regional lymph node metastases in rats exposed to a single dose of 570 rad. ($^{60}$Co $\gamma$-rays) whole body irradiation 24 hours preceding inoculations are shown in Fig. 5 and 6 and are compared with growth in unirradiated recipients. For the first week, the growth curves for Pr

FIG. 6.—Growth curves for primaries (Pr.) and metastases in lymph nodes in unirradiated (closed symbols) and irradiated (open symbols), rats inoculated with $10^6$ or $10^4$ P-388 cells; 6 rats per point, abbreviations as in Fig. 3. Measurements made on day 14 and on day 16 were on two groups of 6 and 3 irradiated rats inoculated with $10^2$ cells and $10^3$ cells respectively, 2 hours after 570 rad. whole body irradiation.

ø ⍙ ⍫ Measurements on day 14.

ø ⍙ ⍫ Measurements on day 16.

and metastases in unirradiated rats were essentially similar in form to those obtained with larger inocula in unirradiated recipients, but due to more rapid growth, somewhat larger tumours resulted in irradiated recipients. Beyond 7 days the rate of growth of Pr decreased at a disproportionate rate in irradiated rats and Pr often decreased in weight when the irradiated rats became terminal and suffered from severe anaemia, loss of weight and widespread growth of pulmonary metastases (*vide infra*). The latter was a principal cause of death during the second week after inoculations of $10^4$ or more cells in rats given WBI. The terminal decrease in growth rate and weight of larger tumours in the irradiated host rat is attributed to nutritional factors and not to the recovery of an immune or some other host defence mechanism, since fewer ($10^2$–$10^3$) cells allowed to grow for longer produced equally large tumours (Fig. 6), and since no comparable decrease in growth rate of lymph metastases (small by comparison with the corresponding Pr) occurred in irradiated rats in which concomitant decreases in growth rate of the larger Pr had taken place.

The most striking difference in tumour growth between irradiated and unirradiated recipients was the rate of growth of metastases following the inoculation of fewer ($10^4$) cells (Fig. 6); irradiation caused lymph node metastases to appear and grow much more rapidly and at rates comparable to the primary. This is attributed to the arrest of immunological reactions after WBI and the associated decrease in the $ED_{50}$ value, which allows growth of a very few newly deposited cells to occur. The curves for enlargement of lymph nodes in unirradiated rats cross those in irradiated animals (Fig. 6). This is principally due to atrophy of normal node tissues produced by WBI but also to the contribution made by hyperplasia to weight of nodes in unirradiated rats. The latter is negligible after WBI since the inoculation of as many as $10^8$ HR tumour cells into the muscle of irradiated rats caused no significant increases in weight of crural and pelvic nodes.

The dose (570 rad.) of WBI used in these experiments allowed > 90% of unchallenged rats to survive beyond 30 days; during the first 7 days weight of the spleen and thymus decreased by > 50% and > 80% respectively, and these losses in organ weight were not reversed during the first 10 days, similar delays being associated with recovery in changes of body weight and anaemia and leucopenia due to WBI. These findings together with data based on $ED_{50}$ determinations and tumour growth rates suggest that immunological defences remained severely attenuated for at least a week after WBI.

*Tumour doubling times*

The doubling time for tumour mass ($T$) was plotted as a function of weight of tumour ($W$). In unirradiated recipients inoculated intramuscularly with $10^6$ cells $T$ was linearly related to $W$ by the relationship,

$$\log T = \mathrm{m} \log W + \mathrm{c}$$

where m and c are constants. Data for smaller tumours produced by subcutaneous growth of $10^6$ Yoshida sarcoma cells in the flank of rats reported by Hirai *et al.* (1968) and our own data for larger P-388 tumours from intramuscular inoculations, were fitted by the same linear relationship (Fig. 7). The slope for growth of $10^7$ cells inoculated into muscle was of the same order of magnitude as for $10^6$ cells, but $10^4$ cells gave a much steeper increase in $T$ with growth of tumour. This is attributed to the more efficient destruction of tumour by immune reactions in an animal challenged with a small inoculum, which takes longer to grow to a tumour of given size, and thereby allows a higher level of immunity to be achieved. Measurements of tumour doubling times after WBI, were necessarily restricted to smaller inocula since larger inocula grew and produced extensive metastases, at an early stage, resulting in nutritional decline and early death of rats. Fig. 7 shows changes in $T$ for growth of Pr and PN metastases produced by $5 \times 10^3$ cells in both unirradiated and irradiated recipients, in an experiment using donor cells from a single rat. After WBI Pr and PN both grew at exponential rates (*i.e.* $T$ constant) until the primary tumour weighed $\sim$ 0·5 g. when $T$ (Pr) increased but the metastases (PN,

FIG. 7. Doubling time for increase in weight of tumour plotted as a function of tumour weight. Figure on left shows data for growth of primary tumours in unirradiated rats produced by intramuscular inocula of $10^4$, $10^6$ and $10^7$ P-388 cells respectively (open symbols); it includes data for growth of $10^6$ Yoshida sarcoma cells inoculated subcutaneously in flanks published by Hirai *et al.* (1968) which is shown by closed circles. Figure on right shows doubling times for growth of primary tumour (Pr.) and pelvic lymph node metastases (PN) produced by $5 \times 10^3$ cells inoculated intramuscularly in unirradiated rats (open symbols) and in irradiated (570 rad. WBI) rats (closed symbols) respectively.

and also UAN and CN) continued to grow at exponential rates while their weights were less than that of the Pr (*i.e.* < 0·5 g.). In unirradiated rats $T$ (PN) increased more rapidly than $T$ (Pr). This may be due to more efficient immunological destruction of tumour cells in the nodes *per se*, but since immunization due to growth of tumour in the rat is time dependent (Fig. 1, *vide supra*) and can cope more efficiently with fewer tumour cells, these factors may also account in part for this difference. However, it was found that when $T$ was expressed as a function of time after inoculation (tumour age $t$) that $T$ (metastasis) increased more rapidly than $T$ (primary). Thus $T$ for both Pr and PN metastases increased at approximately linear rates with time ($t$) after inoculation of tumour cells according to

$$T = k_1 t + k_2$$

where $k_1$ and $k_2$ are constants—a relationship also fitted by the data of Hirai *et al.* (1968) for primary tumours. For 5 × $10^3$ cells inoculated into unirradiated rats values of $k_1$ were 0·55 (primary) and 1·65 (PN metastases); corresponding values for m (*T* expressed as a function of *W*) were 0·91 (primary) and 1·65 (PN metastases).

*Primary and metastasis growth relationships*

The ratio (M/P) for weight of a lymph node metastasis, corrected for normal tissue weight (M), to weight of the primary tumour of origin (P) was calculated for each rat and plotted as a function of age of tumour, for growth ot tumour in unirradiated and irradiated recipients M/P increased at a linear rate (Fig. 8). The rate was somewhat higher for metastases in the more proximally situated pelvic nodes—a difference possibly due to redistributions in flow of lymph and tumour cells produced by progressive nodal involvement. The finding that the rate of increase in M/P with age of primary tumour was less for a small inoculum ($10^4$ cells) than for large inocula ($10^6$–$10^7$ cells) in unirradiated recipients, but not in irradiated

FIG. 8.—Change in ratio (M/P) of weight of lymph node metastases (corrected for normal node weights) to weight of primary tumour, during growth of P-388 tumour in unirradiated and irradiated female rats produced by cell challenges of $10^4$–$10^7$ cells. Values of M/P for pelvic nodes (PN) and upper abdominal nodes (UAN) calculated separately in each rat. Each point represents mean value (±SE) for a group of 5–6 rats. The relationship of M/P to tumour age (*T*) is essentially linear such that M/P = a *T* + b; the same calculated mean values of a (PN) = 0·0295 and a (UAN) = 0·0235 respectively were obtained in irradiated recipients given $10^4$ or $10^6$ cells, and the slopes for PN, UAN were the same in unirradiated recipients given 5 × $10^6$ or $10^7$ cells. In unirradiated recipients which received $10^4$ cells, the rate of increase in M/P with time was significantly less (a (PN) = 0·0140 and a (UAN) = 0·0110 respectively). In 5 experiments, the ratio a (UAN)/a (PN) ≃ 0·79.

recipients, is of particular significance. It is considered that in this allogeneic system immunity destroys smaller tumour deposits of more recent origin more effectively than larger ones, and thereby limits the development and growth of metastases to a greater extent than growth of the primary tumour of origin. After WBI, slopes for M/P corresponding to PN and UAN were not affected by change in the number of cells inoculated over the range $10^2$–$10^6$ cells. In Fig. 9 PN and UAN

FIG. 9.—Data from Fig. 6 for $10^2$–$10^6$ inocula in irradiated recipients and $5 \times 10^6$–$10^7$ inocula in unirradiated recipients respectively has been pooled. Regression lines of the same slope (a) according to the equation $(M/P) = a\,T + c$ have been arbitrarily fitted for growth of pelvic node metastases (PN) in irradiated and unirradiated recipients respectively, and similarly for growth of tumour in upper abdominal nodes (UAN). The regressions are given by:

PN (irradiated recipients) $M/P = 0{\cdot}029\,T - 0{\cdot}100$
(unirradiated recipients) $M/P = 0{\cdot}029\,T - 0{\cdot}017$
UAN (irradiated recipients) $M/P = 0{\cdot}017\,T - 0{\cdot}075$
(unirradiated recipients) $M/P = 0{\cdot}017\,T - 0{\cdot}062$

ratios have been pooled for $10^2$–$10^6$ cells inoculated in irradiated rats and for $10^6$–$10^7$ cells in unirradiated rats respectively. The two sets of data for irradiated and unirradiated recipients have been fitted by linear relationships with the same slope, 0·029 (PN) and 0·017 (UAN) respectively. The rate of increase in the ratio M/P with age of tumour provides a useful quantitative approach to the study of kinetic aspects of metastatic dissemination and would seem valuable for determining the effects of physical, chemical and biological treatments, specifically concerned with the control of such spread. Changes are produced by age and size of tumour in the contents of blood, oedema and necrosis and this affects the interpretation of changes in M/P—particularly early after inoculation when disparity in size between Pr and M is greatest and the onset of haemorrhage and oedema cause greater increases in weight of the more advanced primary tumour.

In rats sacrificed 7 days after inoculating $10^2$–$2 \times 10^7$ cells, tumour weights (Pr and PN, UAN and CN metastases) were plotted on a log–log scale as a function of the number (N) of cells inoculated, for unirradiated and irradiated recipients respectively (Fig. 10). After initial slow increases in tumour weights, the relation-

FIG. 10.—Relationship between number of tumour cells inoculated intramuscularly and weight of primary tumour, lymph node metastases and number of lung metastases in (A) irradiated and (B) unirradiated recipient rats, killed 7 days after inoculation. Values for lung metastases enumerated on pleural surfaces (200 the maximum number scored) are shown for individual rats. Each point for mean tumour weight based on 6 rats; each standard error of mean (vertical line on symbol) shown for group A measurements only.

ships became linear, beyond "threshold" values of N $\simeq 10^3$ (irradiated) and N $\simeq 10^5$ (unirradiated recipients). The slopes of the linear regions for Pr and its metastases in immunologically suppressed and intact hosts, were calculated and found to approximate to a common value of 0·308. The difference in N of 2–3 log values between irradiated and unirradiated recipients for the onset of linear increases in tumour weight, corresponds to the difference in $ED_{50}$ values.

Associated changes in the ratio M/P (7 days), expressed as a function of size of inoculum (N), were obtained in young female recipient rats (90–100 g. body weight) using tumour cells harvested from a single donor rat (Fig. 11). A very large

FIG. 11.—Showing effects of increase in number of P-388 cells inoculated on (a) ratio M/P for node metastases (b) body weight of inoculated rats during growth of tumour and (c) weight of spleen and thymus (expressed per unit body weight) in unirradiated (open symbols) and irradiated (closed symbols) recipients. Data shown for changes in body weight and weights of spleen and thymus, are confined to 90–100 g. rats which were killed 8 days after inoculation. Data for M/P pooled from a series of experiments on rats varying more widely in age; most were killed after 7 days but some (given smaller inocula) after 8 days.

challenge dose ($\sim 10^7$ cells) was required to raise the values of M/P for PN and UAN metastases in unirradiated recipients to equal those in irradiated recipients. This represents the threshold in cell number required to initiate growth in this tumour host system against which immunological defences mounted by the host against spread of tumour to the nodes becomes insignificant in the control of such spread or in reducing the rates of growth of tumour at the primary site and in related nodes.

### *Metastases to lungs and other organs*

*Lungs.*—Most rats inoculated with $10^4$ or more P-388 cells developed multiple macroscopic pulmonary metastases. In lungs with 100 or more enumerated surface nodules lung weights were increased and as much as three- to four-fold heavier than normal in terminal rats.

TABLE I.—*Incidence of Rats with Pulmonary Metastases, and Numbers of Pulmonary Metastases Scored in Unirradiated and Irradiated Female Rats at Various Times after the Intramuscular Inoculation of P-388 Tumour Cells*

| N | T | WBI (+, −) | I | PM |
|---|---|---|---|---|
| $10^2$ | 8 | + | 6 | 6, 20, 4, 8, 6, 20 |
| | 14 | + | 6 | 5, 26, 3 and > 100 (3 rats) |
| $10^3$ | 8 | + | 6 | 14, 8, 27, 1, 5, 37 |
| | 13 | + | 6 | 70, 5 and > 100 (4 rats) |
| $10^4$ | 7 | − | 0 | — |
| | 8 | + | 6 | 15, 25, 16, 5, 16, 20 |
| | 10 | − | 3 | 3, 3, 4 |
| | 10 | + | 6 | 10, 27, 14, 15, 39, > 100 |
| | 11 | + | 6 | > 100 (6 rats) |
| | 14 | − | 2 | 2, 17 |
| $10^5$ | 7 | − | 3 | 2, 5, 1 |
| | 7 | + | 4 | 4, 1, 4, 2 |
| | 8 | + | 6 | 107, 161, > 200 (4) |
| $10^6$ | 4 | − | 1 | 2 |
| | 7 | − | 0 | — |
| | 7 | + | 6 | 66, 48, 85, 18, 6, 43 |
| | 10 | − | 6 | 9, 3, 1, 2, 4, 1 |
| | 9 | + | 6 | > 200 (6 rats) |
| $10^7$ | 7 | − | 3 | 5, 1, 93 |
| | 7 | − | 4 | 2, 2, 2, 57 |
| | 7 | + | 6 | > 200 (6 rats) |

*Abbreviations:* Number of P-388 cells inoculated N, time (days) after inoculation T, whole body irradiation WBI given 2–24 hours preceding inoculation (+) or omitted (−); number of rats which developed pulmonary metastases I in each group consisting of 6 rats; number of metastases scored in lungs of each rat on macroscopic examination of freshly excised lungs or of lungs fixed in Bouin's fluid PM.

Table I and Fig. 10 show the number of pleural metastases enumerated in lungs of unirradiated and irradiated rats inoculated with $10^2$–$10^7$ P-388 cells. The incidence rose with the number of cells inoculated and with time after inoculation and was much higher after WBI. Both unirradiated and irradiated rats with pulmonary metastases, showed the presence of tumour cells in ventricular blood and in irradiated rats with massive pulmonary involvement, tumour cells were often the commonest nucleated cell present. Pulmonary metastases were absent or few in number in rats killed with early abdominal lymph node metastases and only appeared in large numbers of rather uniform size after more widespread metastases in lymph nodes had occurred and irrespective of size attained by the primary tumour. Fig. 10 shows that whole body irradiation reduced the number of inoculated cells required to produce an arbitrarily selected mean incidence of 50 pulmonary metastases in rats by approximately 2·5 log values—a difference similar to that for $ED_{50}$ values in irradiated and unirradiated rats. Approximately $10^6$ cells in unirradiated, and $10^3$–$10^4$ cells in irradiated rats induced the same number of pulmonary metastases. This suggests that irradiation causes a similar degree of immunological attenuation in the lungs as in other tissues (primary site and lymph nodes), and that the incidence of lung metastases simply represents the degree of " spill over " of tumour from the lymphatic system into the circulation and lungs.

*Heart and kidneys.*—Rats with advanced lymph node and lung metastases, frequently showed growing metastases in the myocardium, particularly in ventricular musculature. Advanced upper abdominal lymph node metastases had frequently invaded adjacent kidneys and other organs by direct infiltration. In rats terminal with widespread pulmonary metastases, the kidneys (and other organs) also frequently showed multiple small subcapsular metastases similar to those described for lungs, and which appeared to have arisen by exfoliation from lung deposits and haematogenous dissemination.

*Spleen ana thymus.*—An example of the changes in weight of spleen and thymus in unirradiated and irradiated recipients is shown in Fig. 11. Unirradiated recipients inoculated with $10^7$ tumour cells developed splenomegaly ($\sim 15\%$ increase in weight) accompanied by decrease ($\sim 40\%$) in weight of thymus, and reduction ($\sim 30\%$) in the rate of growth of the rat during the first 7–10 days. WBI (570 rad.) caused $\sim 50\%$ decrease in weights of spleen and thymus respectively and body growth was decreased by $\sim 25\%$. When rats, after WBI, were inoculated with P-388 cells body growth was markedly affected, and inocula of $10^7$ cells caused rats to lose weight at $> 1$ g. per day. Irradiated rats showed the same pattern of splenic enlargement and thymic atrophy due to tumour growth as unirradiated rats, the changes being superimposed on the atrophy of these tissues produced by WBI itself (Fig. 11). The cause for these opposite changes in spleen and thymus weight is not clear. Thymus atrophy may be due to stress, aside from irradiation damage, and seems to parallel changes in body weight. Increases in spleen weight appears largely due to growth of tumour in this organ, being proportional to the number of cells inoculated and occurring more rapidly in irradiated rats; microscopically, the spleens showed discrete solid foci of tumour cell growth present mainly in white pulp. However, whether tumour growth accounts entirely for the changes in spleen weight has not been determined.

### DISCUSSION

Relatively few allogeneic or syngeneic transplantable tumours are available for quantitative experimental investigations of metastatic spread (Kim, 1970) and to the role played by immunological reactions in growth of metastases. Although several experimental tumours produce metastases these may not develop in a particular organ with sufficient regularity to study the kinetic relationships between their growth and that of the primary tumour of origin in the same animal. P-388 sarcoma, an allogeneic, rapidly growing tumour host system in the rat, was chosen principally to investigate these aspects, since it could be grown as a solid tumour which metastasized along lymphatic pathways to lymph nodes and lungs, and the recipient rat showed immunological resistance to tumour growth which could either be suppressed or increased by suitable treatment of recipients before challenge with the tumour. Growth of the tumour as an ascites for passaging also facilitated accurate enumeration of cells for inoculations and assays.

By weighing the primary tumour and individual lymph node metastases associated with its lymphatic spread in animals killed at various times after inoculation with a known number of tumour cells, growth curves were obtained for primary and metastases and compared. The enumeration of lung metastases and increases in lung weight in such animals provided further measures of metastatic behaviour. The rapid rate of growth of P-388 sarcoma and its metastases also allowed suitable measurements of growth to be made under conditions of immunological suppression by sub-lethal whole body irradiation of the host before significant immunological recovery took place and thereby provided an immunological situation more closely allied to a syngeneic or autochthonous tumour-host system.

Certain characteristics of P-388 growth in tissues, shared by other experimental tumours, present some difficulties to quantitative studies and the tumour appears to differ somewhat in form and nutrition, from various spontaneous, and particularly less rapidly growing, cancers in man and animals. Its growth is essentially infiltrative and fails to stimulate a commensurate growth of stroma from normal tissues including new vessel formation (angiogenesis) and formation of a tumour capsule. Consequently, tumour nutrition largely comes to depend on the resources of existing tissues being invaded by the growing tumour. The latter progressively destroys normal capillaries and blood vessels in these supportive normal tissues and this causes haemorrhage into the tumour, and oedema. Nutrition and oxygenation become inadequate for tumour growth and rapid tumour necrosis develops. Inadequate nutrition and progressive necrosis appear largely responsible for the curvilinear form of tumour growth and for increases in tumour doubling time for both primary and metastasis. However, incompatibility between tumour and host (homograft-reactions) and the progressive enhancement of these reactions produced by growth of tumour must also decrease growth rates since doubling times remain constant for longer in immunologically attenuated (irradiated) hosts. Mayneord (1932) proposed a dynamic model of tumour growth based on data provided by the Jensen sarcoma in the rat, in which the growth pattern was governed by a linear law (tumour dimensions with time) as opposed to an exponential relationship, and proliferative growth was confined to an outer narrow surface zone of the tumour. The data obtained by Hirai *et al.* (1968) for Yoshida sarcoma was found to fit the Mayneord model, as did our own data for both primary and node metastases from P-388 sarcoma in unirradiated (immunologically intact) rats. The data of Hewitt and Blake (1968) for a slow growing syngeneic murine tumour also appears to fit the Mayneord model. However, the rate of growth of P-388 sarcoma in the immunologically intact (incompatible) host depended largely on the size of the challenge dose and the effect of the latter on the rate at which immunity to growth of tumour increased with time. Thus we prefer to analyse tumour doubling time ($T$) in terms of tumour size ($W$) rather than age of tumour ($t$). $T$ and $W$ were linearly related on a log–log plot, but $T$ increased more rapidly if fewer cells were inoculated. Since smaller inocula take longer than larger inocula to grow to a tumour of the same mass, smaller inocula cause more prolonged stimulation of immunological functions and cause a higher level of immunity to develop. For lymph node metastases, $T$ rose more rapidly than for the primary tumour of origin. This suggests that more efficient immunological defences develop in the node, but since the disparity in size between primary and secondary tumours would also affect the efficacy of an immune reaction, this needs to be taken into account. WBI, which suppresses early immunological reactions to a tumour allograft to insignificant proportions in the P-388 system and also in the Ehrlich tumour (van den Brenk, 1961), caused growth of both the primary P-388 tumour and its metastases to approximate to an exponential rate of growth during the initial stages when relatively little tumour necrosis had occurred. This finding suggests that immunological incompatibility plays a significant and possibly major role in causing curvilinear forms of tumour growth and increases in doubling times, particularly when this is shown by *early* infiltrative growth of solid tumours such as poorly differentiated rat sarcomata. The nutrition made available by existing vasculature and stroma, even after the latter has been irradiated during whole body exposure of 570 rad., appears adequate to support exponential rates of growth for a time and supportive angiogenesis appears unnecessary to supplement nutritive requirements at this stage. The factors responsible for linear rates of tumour growth, expressed in terms of tumour dimensions with ageing, in syngeneic tumours, which show no significant immunological incompatibility (Hewitt and Blake, 1968) and possibly in spontaneous tumours, may be related to cell losses resulting from cellular differentiation and to the " controlling " effect imposed on tumour cell growth and differentiation by the stimulation of stroma by a tumour as a co-ordinated trophism. Pressure produced by expansive growth, particularly in more well encapsulated tumours, may be another factor, but no satisfactory explanation accounts for changes in growth rate of solid tumours, which encompasses cellular kinetics, nutrition, immunity and a variety of other pathophysiological factors. Indeed it seems rather remarkable that the many positive and negative factors which interact to determine tumour growth rates allow any general quantitative law of growth to be applicable. Some further difficulties clearly arise in interpreting curves of tumour growth after whole body irradiation of recipients. The irradiation potentially both facilitates growth of tumour by inhibiting immunity and restricts it by reducing the proliferative capacity of host tissues to provide a nutritive stroma. For P-388 sarcoma the ratio M/P (mass of metastasis relative to mass of primary) increased at a linear rate with tumour age. This relationship was independent of size of inoculum and the state of immunity. The rate of increase in M/P was also relatively constant and was reduced only by inoculating fewer cells in immunologically intact animals when the immunological reactions were efficient in destroying less advanced growing tumour. Since rates of increase in M/P were the same for growth of tumour in unirradiated rats given large inocula as in irradiated rats challenged with few (or many) cells, this suggests that the effects of whole body irradiation on the development and growth of metastases is limited to immunological effects. WBI does not appear to affect significantly other physiopathological mechanisms, local or general, associated with the production and growth of metastases such as exfoliation, transfer, arrest and nutrition of cells in organs. Sublethal WBI causes marked metabolic changes associated with cellular destruction and other physiological changes in organs and tissues, which might be expected to modify the growth of unirradiated tumour cells deposited in these tissues, but the data obtained fail to support this view.

The progressive increase in immunity produced by growth of tumour in the rat enables unirradiated rats to survive with large and progressively growing primary tumours or with isolated large growing metastases, by destroying newly deposited cells and thereby preventing other metastases developing. It also determines survival of rats in which nodes may remain chronically enlarged due to the dual presence of newly formed and growing and degenerating tumour foci. Similarly, animals which develop very large primaries may remain otherwise healthy and appear free of metastases since a high degree of immunity develops which prevents the establishment and growth of further metastases. Such clinical situations—not so different from those experienced from time to time in spontaneous human cancer and its metastases—never occurred after immunological attenuation from WBI. Experience with local irradiation of large growing primary tumours in rats not pre-treated with WBI showed that such locally advanced tumours were relatively readily cured by modest dosages (unpublished results; see also Fig. 1).

Furthermore, such local irradiation treatments did not reduce immunity enhanced by previous growth of tumour, since the incidence of subsequent metastasis did not increase and the treated rats were subsequently shown to be resistant to further challenges of $10^6$ or more P-388 cells.

### REFERENCES

Apffel, C. A., Arnason, B. G. and Peters, J. H.—(1966) *Nature, Lond.*, **209**, 694.

van den Brenk, H. A. S.—(1961) *Br. J. Cancer*, **15**, 61 and 798.

Hewitt, H. B. and Blake, E. R.—(1968) *Br. J. Cancer*, **22**, 808.

Hirai, E., Niibe, H., Tobe, T., Kawashima, K. and Yonome, I.—(1968) *Nippon Acta radiol.*, **28**, 986.

Kim, U.—(1970) *Science, N.Y.*, **167**, 72.

Mayneord, W. V.—(1932) *Am. J. Cancer*, **16**, 841.

Yoshida, T.—(1949) *Gann*, **40**, 1.—(1952) *J. natn. Cancer Inst.*, **12**, 947.